# Current insights into the effects of cationic biocides exposure on *Enterococcus* spp.

**DOI:** 10.3389/fmicb.2024.1392018

**Published:** 2024-06-25

**Authors:** Ana P. Pereira, Patrícia Antunes, Luísa Peixe, Ana R. Freitas, Carla Novais

**Affiliations:** ^1^UCIBIO-Applied Molecular Biosciences Unit, Laboratory of Microbiology, Department of Biological Sciences, Faculty of Pharmacy, University of Porto, Porto, Portugal; ^2^Associate Laboratory i4HB - Institute for Health and Bioeconomy, Faculty of Pharmacy, University of Porto, Porto, Portugal; ^3^Faculty of Nutrition and Food Sciences, University of Porto, Porto, Portugal; ^4^1H-TOXRUN, One Health Toxicology Research Unit, University Institute of Health Sciences, CESPU CRL, Gandra, Portugal

**Keywords:** *Enterococcus*, biocides, quaternary ammonium compounds, biguanides, susceptibility, One Health

## Abstract

Cationic biocides (CBs), such as quaternary ammonium compounds and biguanides, are critical for controlling the spread of bacterial pathogens like *Enterococcus* spp., a leading cause of multidrug-resistant healthcare-associated infections. The widespread use of CBs in recent decades has prompted concerns about the potential emergence of *Enterococcus* spp. populations exhibiting resistance to both biocides and antibiotics. Such concerns arise from their frequent exposure to subinhibitory concentrations of CBs in clinical, food chain and diverse environmental settings. This comprehensive narrative review aimed to explore the complexity of the *Enterococcus*’ response to CBs and of their possible evolution toward resistance. To that end, CBs’ activity against diverse *Enterococcus* spp. collections, the prevalence and roles of genes associated with decreased susceptibility to CBs, and the potential for co- and cross-resistance between CBs and antibiotics are reviewed. Significant methodological and knowledge gaps are identified, highlighting areas that future studies should address to enhance our comprehension of the impact of exposure to CBs on *Enterococcus* spp. populations’ epidemiology. This knowledge is essential for developing effective One Health strategies that ensure the continued efficacy of these critical agents in safeguarding Public Health.

## 1 Introduction

The use of cationic biocides (CBs) is critical to control and prevent the dissemination of bacterial pathogens in the most diverse environments (Wales and Davies, 2015; Fox et al., 2022). They have been a cornerstone in the improvement of hygienic practices, preventing infections and, consequently, reducing the need for antibiotic use (Wales and Davies, 2015; Fox et al., 2022). The global antiseptics and disinfectants market, including CBs, has been continuously growing and is expected to reach $13.3 billion by 2028, which is almost double the market size of $7.5 billion in 2020 (Zion Market Research, 2021). Thus, over the last decades, the extensive use of CBs and consequent high exposure of bacteria to these compounds has been raising concerns about the possibility of selection of strains resistant either to these or other antimicrobials, as antibiotics (Moore et al., 2008). The assessment of the impact of biocides in the evolution of *Enterococcus* populations is of current concern, as this is one of the leading multidrug-resistant (MDR) healthcare-associated pathogens worldwide (Guzman Prieto et al., 2016; Garcia-Solache and Rice, 2019). Among the more than 60 validated *Enterococcus* species,^1^ *Enterococcus faecalis* and *Enterococcus faecium* are the two predominantly implicated in human opportunistic infections and are among the most prevalent in the human gut microbiota (Guzman Prieto et al., 2016; Garcia-Solache and Rice, 2019; Zaheer et al., 2020). They are intrinsically resistant to a broad spectrum of antibiotics and have rapidly acquired resistance to other critical ones, particularly to ampicillin or vancomycin among *E. faecium* (Guzman Prieto et al., 2016; Garcia-Solache and Rice, 2019; Freitas et al., 2021). Indeed, vancomycin-resistant *E. faecium* are categorized as high priority on the World Health Organization priority pathogens list for research and development of new antibiotics, causing infections with limited treatment options and associated with high mortality and health care costs (Guzman Prieto et al., 2016; Garcia-Solache and Rice, 2019; Freitas et al., 2021; World Health Organization [WHO], 2024). They are easily spread between patients and across their surrounding hospital environment through fecal contamination of the hands of patients, the healthcare staff and visitors, and of the medical equipment or other inanimate surfaces (Arias and Murray, 2012; Correa-Martinez et al., 2020). Furthermore, due to their remarkable ability to survive harsh conditions, such as nutrient scarcity or desiccation, *Enterococcus* spp. might remain on these contaminated surfaces for extended periods, even years (Arias and Murray, 2012; Dancer, 2014; Suleyman et al., 2018; Gaca and Lemos, 2019; Correa-Martinez et al., 2020). Effective antisepsis and disinfection practices are, therefore, crucial to break the chain of transmission, prevent spread of these microorganisms in the hospital environment and potential life-threatening MDR infections. *Enterococcus* spp. exposure to CBs, including at subinhibitory concentrations, extends beyond human clinical settings, as they are part of the natural microbiota of plants, soil, and the human and animals’ gastrointestinal tract, and cause animal infections (Gnanadhas et al., 2013; Guzman Prieto et al., 2016; Maillard, 2018; Garcia-Solache and Rice, 2019; McCarlie et al., 2020; Zaheer et al., 2020; Fox et al., 2022). Therefore, CBs also play a key role in the control of their transmission in the veterinary, food chain (e.g., food industry, farms), or human domestic contexts (Gnanadhas et al., 2013; Guzman Prieto et al., 2016; Maillard, 2018; Garcia-Solache and Rice, 2019; McCarlie et al., 2020; Zaheer et al., 2020; Fox et al., 2022). In addition, *Enterococcus* spp. are exposed to CBs in sewage, aquatic systems, and soil/sediments, resulting from domestic, hospital, and industrial discharges (Matsushima and Sakurai, 1984; European Medicines Evaluation Agency [EMEA], 1996a; Lucas, 2012; Cowley et al., 2015; Tezel and Pavlostathis, 2015; Ostman et al., 2017; Environment and Climate Change Canada [ECCC], 2019; Pereira and Tagkopoulos, 2019; Pati and Arnold, 2020).

Among CBs, the most common and to which the susceptibility of *Enterococcus* spp. has been increasingly studied in the last years, are the quaternary ammonium compounds (QACs) and the biguanides (Moore et al., 2008; Fox et al., 2022; Maillard and Pascoe, 2024). However, the dispersed and sometimes contradictory information, coupled with limitations in study designs impacting their general conclusions, underscores the need for a comprehensive literature review to establish a standpoint on current data and address existing research gaps.

Here, we reflected on CBs’ activity against *Enterococcus* spp. from different sources, geographical regions and years, while taking into consideration the current challenges associated with the study of biocide susceptibility. Moreover, we explore the evolution of decreased susceptibility to CBs reported for particular enterococcal populations of diverse epidemiological backgrounds as well as discuss the outcomes of *in vitro* exposure of *Enterococcus* spp. to subinhibitory concentrations of CBs. The prevalence of genes with a known or predicted role on CBs susceptibility in *Enterococcus* spp. and their confirmed or possible link to decreased susceptibility phenotypes are also reviewed, as well as the potential risk of co- and cross-resistance between CBs and antibiotics. Finally, this review highlights the methodological and knowledge gaps that need to be addressed in future research to better understand the implications of CBs use on *Enterococcus* spp. evolution and ultimately contribute to the development of appropriate interventions in diverse sectors highly exposed to CBs.

## 2 Cationic biocides

**FIGURE 1:**
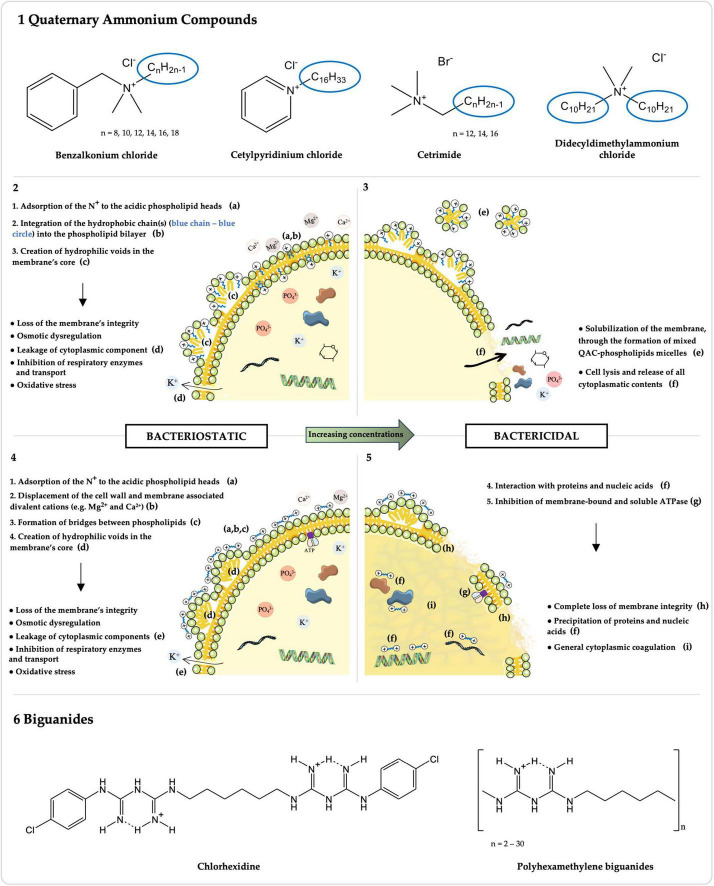
Chemical structures and mechanisms of action of Quaternary Ammonium Compounds (QACs) and Biguanides. The chemical structures of the most common QACs (1) and biguanides (6), whose activity against *Enterococcus* spp. has been studied, are presented along with their respective mechanisms of action for bacteriostatic (2: QACs; 4: biguanides) and bactericidal (3: QACs; 5: biguanides) concentrations. Molecules were drawn using ChemDraw v16.0 (https://revvitysignals.com/products/research/chemdraw). The figure was partly generated using Servier Medical Art, provided by Servier, licensed under a Creative Commons Attribution 3.0 unported license.

Cationic biocides (CBs) are broad-spectrum antimicrobial compounds widely employed in disinfectants, antiseptics, and preservatives (Fox et al., 2022). These agents have been used in clinical and domestic settings, the food industry, agriculture, and other sectors since the 1930s (Gilbert and Moore, 2005; Moore et al., 2008). CBs antibacterial mechanism of action primarily targets the negatively charged cytoplasmic membrane (Salton, 1951; Hugo and Longworth, 1964, 1966; Denton, 1991; Gilbert and Moore, 2005; Maillard and Pascoe, 2024). However, the specific interaction of each CB with the membrane and subsequent concentration-dependent bacteriostatic or bactericidal mechanisms differ between the chemically diverse compounds (Figure 1) (Salton, 1951; Hugo and Longworth, 1964, 1966; Denton, 1991; Gilbert and Moore, 2005; Maillard and Pascoe, 2024). Indeed, these membrane-active agents fall into different classes with various chemical structures (Figure 1), whose intrinsic properties influence the CB’s activity (Salton, 1951; Hugo and Longworth, 1964, 1966; Denton, 1991; Gilbert and Moore, 2005; Maillard and Pascoe, 2024).

### 2.1 Quaternary ammonium compounds

Over the past decades, and particularly in recent years, the use of QACs has been increasing in diverse fields of application (Gerba, 2015; Buffet-Bataillon et al., 2016; Hora et al., 2020). Their general chemical structure is N^+^R_1_R_2_R_3_R_4_ X^–^, where R represents hydrogen atoms, alkyl or aryl groups, and X represents an anion, commonly Cl^–^ or Br^–^ (Figure 1-1; Buffet-Bataillon et al., 2012; Gnanadhas et al., 2013). The antimicrobial activity of QACs correlates with the n-alkyl chain length, which, against Gram-positive *Staphylococcus aureus*, is optimal at n = 14 (Daoud et al., 1983; Gilbert and Al-Taae, 1985). This is because QACs’ hydrophobic tail(s) directly interact with the cytoplasmagic membrane (Figure 1-2,3; Salton, 1951; Ioannou et al., 2007). The mechanism of action of QACs has been also studied against other Gram-positive bacteria, namely *Enterococcus* spp. (Salton, 1951; Ioannou et al., 2007). After the adsorption of the positively charged quaternary nitrogen to the acidic phospholipid heads in the membrane, the hydrophobic chain(s) interdigitates into the hydrophobic bilayer, creating hydrophilic voids in the membrane’s core (Figure 1-2; Salton, 1951; Gilbert and Moore, 2005; Ioannou et al., 2007). This results in a loss of the membrane’s physical and ionic integrity, with leakage of cytoplasmic components, osmotic dysregulation, inhibition of respiratory enzymes and transport, and oxidative stress (Figure 1-2; Salton, 1951; Gilbert and Moore, 2005; Ioannou et al., 2007). With increasing concentrations, QACs have a bactericidal action by solubilizing the hydrophobic membrane components, with the formation of mixed QAC-phospholipids micelles, lysing the cell and consequently releasing all cytoplasmatic contents (Figure 1-3; Salton, 1951; Gilbert and Moore, 2005; Ioannou et al., 2007).

A wide variety of QACs has been formulated over the years with increasing antimicrobial efficacy and improved activity in adverse conditions (e.g., anionic residues, hard water) (Gerba, 2015; Rutala et al., 2019; Belter et al., 2022). Among the most broadly used QACs, to which susceptibility of *Enterococcus* spp. has been studied, are benzalkonium chloride (BC), cetylpyridinium chloride (CPC), cetrimide (CE), and didecyldimethylammonium chloride (DDAC) (Figure 1-1 and Supplementary Table 1; Buffet-Bataillon et al., 2012).

Benzalkonium chloride (BC) is a widely used mixture of n-alkyl-dimethyl-benzyl-ammonium chlorides, with variable n-alkyl chain lengths, typically ranging from 8 to 18 carbons (Figure 1-1; Gilbert and Moore, 2005; Buffet-Bataillon et al., 2012; Belter et al., 2022). It has been used since the 1930s and its extensive applications span from personal care products (e.g., mouthwashes, shampoos and body lotions), to disinfectants and antiseptics in household, industrial, agricultural, and clinical environments, or as mitigators of microbial metal corrosion within oil pipelines and cooling water systems, with concentrations ranging from 20 mg/L, in ophthalmologic formulations, to 20,000 mg/L, in wood preservation products (Liu et al., 2017; Kampf, 2018a; Pereira and Tagkopoulos, 2019; Short et al., 2021; Kheljan et al., 2022; Wang et al., 2023). BC’s concentrations of 100–3,000 mg/L are used for healthcare and household antisepsis and surface disinfection (Kampf, 2018a; Fox et al., 2022).

Other QACs include cetylpyridinium chloride (CPC), also known as 1-hexadecylpyridinium chloride, corresponding to the chlorine salt of a positively charged pyridine bonded to a hexadecane lipophilic chain, and cetrimide (CE), which consists in a mixture of tetradecyltrimethylammonium, dodecyltrimethylammonium, and hexadecyltrimethylammonium bromides (Figure 1-1; Council of Europe, 2019; Mao et al., 2020; Lv et al., 2023; Okeke et al., 2023). CPC, whose antimicrobial activity was first described in 1939, has been used for decades in dentistry, being predominantly found in over-the-counter oral hygiene products, such as mouthwashes, toothpastes, and sprays, at 30–3000 mg/L, for the prevention and control of oral infections (Mao et al., 2020; Komine et al., 2021; Takeda et al., 2022; Lv et al., 2023; Setiawatie et al., 2023). In addition, it is approved by regulatory agencies of several countries including the USA, but not the European Union, for the sanitization of poultry carcasses in poultry processing plants, at concentrations of ≤1% (Beers et al., 2006; Waldroup et al., 2010; Safe Foods Corporation, 2019; FSIS - U.S. Food Safety and Inspection Service - Department of Agriculture, 2023). CE, in use since 1942, serves as a topical antiseptic for cleaning the skin, wounds and minor burns, in dentistry, and for the treatment of nappy rash, acne and seborrheicitis, in concentrations between 1,000 and 30,000 mg/L (European Medicines Evaluation Agency [EMEA], 1996b).

Didecyldimethylammonium chloride (DDAC), a twin QAC developed in the 1960s, features two long-chain alkyl groups and two methyl substituents bonded to the positively charged nitrogen, along with the negatively charged chloride anion (Figure 1-1; Kampf, 2018b; Belter et al., 2022). DDAC finds applications in antiseptics and disinfectants used in clinical, food chain, and domestic environments, in laundry, agricultural tools and vehicles, in swimming pools and water displays, and various indoor and outdoor hard surfaces (e.g., walls, floors), with concentrations ranging from 200 to 12,000 mg/L (Schwaiger et al., 2014; Kampf, 2018b; Fox et al., 2022).

QACs may leave residues on treated surfaces and in the environment as they are photolytically stable and have long half-lives (e.g., >150 days in pH ≥ 5) (Dizman et al., 2004; Mousavi et al., 2013; Kampf, 2018a,b). They have been found in some types of food including fruits and nuts, vegetables or dairy products (up to 14.4 mg/kg), possibly through contact with disinfected surfaces (European Food Safety Authority [EFSA], 2013; Díez et al., 2016; EURL-SRM - EU Reference Laboratory for Pesticides Requiring Single Residue Methods, 2016). Also, like most trace contaminants, QACs are not completely removed through wastewater treatment, being released into the environment and found in sewage and surface waters in different concentrations (0.0078 μg/L to 6 mg/L) (Tezel and Pavlostathis, 2015; Zhang et al., 2015; Ostman et al., 2017; Pereira and Tagkopoulos, 2019; DeLeo et al., 2020; Kim et al., 2020; Pati and Arnold, 2020). QACs are considered “very toxic to aquatic life with long-lasting effects” by the European Chemicals Agency (ECHA) (European Chemicals Agency [ECHA], 2023a,b). Moreover, QACs are highly biodegradable under aerobic conditions and known to adsorb strongly to the negatively charged surfaces of sludge, soil and sediments, because of their positive charge, interfering with their bioavailability and enabling the fluctuation of QACs’ concentrations that can impact local microbiota (Tezel and Pavlostathis, 2015; Zhang et al., 2015; Kampf, 2018a; DeLeo et al., 2020). Further investigation is needed to determine the influence of other factors promoting the environmental persistence of QACs, such as the emergent micropollutants like microplastics, to which QACs may potentially bind (Kim et al., 2022). While QAC disinfectants have historically been viewed as having low toxicity to humans, recent studies on human and mouse cell lines have shown that chronic exposure can cause inflammation, disrupt mitochondrial function, alter estrogen signaling, and inhibit cholesterol synthesis. Human exposure to QACs likely occurs via dermal contact, inhalation of aerosolized droplets, and ingestion in water and food, highlighting the need for further research, especially in light of the increased use during the COVID-19 pandemic (Hrubec et al., 2021; Frantz, 2023).

### 2.2 Biguanides

Biguanides correspond to a class of compounds that carry the functional moiety HN(C(NH)NH_2_)_2_ (Figure 1-6), comprising antidiabetic (e.g., metformin), antimalarial (e.g., proguanil) and other antimicrobial compounds, some of which are included in the WHO List of Essential Medicines (Gilbert and Moore, 2005; Grytsai et al., 2021; Kathuria et al., 2021; World Health Organization [WHO], 2023). The activity of the two most common biguanide biocides, chlorhexidine digluconate (CHX) and polyhexamethylene biguanides (PHMB), the latter also known as polihexanide (Jones and Joshi, 2021; Kathuria et al., 2021), against *Enterococcus* spp. has been evaluated over the years, particularly for CHX (Figure 1-6 and Supplementary Table 1).

CHX is a bisbiguanide with extensive applications in the human and veterinary healthcare contexts as a hand, surgical site or wound antiseptic, and as a surface and instrument disinfectant, due to its broad-spectrum activity and long-lasting residual activity when comparing to other biocides (Williamson et al., 2017; Kampf, 2018c; Sommer et al., 2019; Fox et al., 2022). Moreover, CHX daily bathing of intensive care unit patients has been increasingly adopted in order to reduce colonization and infection by MDR bacteria, such as vancomycin-resistant *Enterococcus* (Climo et al., 2009; Popovich et al., 2012; Mendes et al., 2016; Lowe et al., 2017; Williamson et al., 2017; Tien et al., 2020). Similarly, CHX is broadly used in household antiseptics, especially in oral, pharmaceutical and handwashing products, disinfectants, and preservatives (e.g., cosmetics and personal care products), as well as in diverse industries (e.g., antiseptic for food handlers and in paper products such as tissues or wall paper) (Kampf, 2018c). In-use concentrations range from 25 to 100 mg/L for preservation to 500–40,000 mg/L for antisepsis and disinfection purposes (Maillard, 2005; Kampf, 2018c; Fox et al., 2022). CHX’s chemical structure consists in a symmetric bisbiguanide with two chloroguanide groups connected by a hydrophobic hexamethylene chain (Figure 1-6; Cieplik et al., 2019; Kathuria et al., 2021). Its antibacterial mechanism of action has been studied among Gram-positive and Gram-negative bacteria, namely *S. aureus* and *Escherichia coli*, respectively (Hugo and Longworth, 1964, 1966; Denton, 1991). A key distinction between bisbiguanides and QACs’ mechanisms of action lies in the solubilization of the hydrophobic regions (Figure 1). Whilst the hydrophobic chain of QACs integrates into the hydrophobic core of the cytoplasmatic membrane, CHX’s, being only 6 carbons long, is not able to do so (Gilbert and Moore, 2005). Instead, as a bacteriostatic, the two positively charged biguanide groups of CHX displace the cell wall and cytoplasmatic membrane associated divalent cations (e.g., Mg^2+^ and Ca^2+^) and associate to the then exposed anionic sites, forming bridges between pairs of adjacent phospholipids (Figure 1-4; Hugo and Longworth, 1964, 1966; Denton, 1991; Gilbert and Moore, 2005). This interaction disturbs the membrane’s fluidity, osmoregulation and metabolism, with increased permeability and leakage of low molecular weight cytosolic components (e.g., potassium ions), and inhibition of transport and cellular respiration (Figure 1-4; Hugo and Longworth, 1964, 1966; Denton, 1991; Gilbert and Moore, 2005). At higher concentrations, CHX has a bactericidal mechanism of action through the complete loss of cytoplasmatic membrane integrity and, ultimately, the precipitation of proteins and nucleic acids and general cytoplasmic coagulation (Figure 1-5; Hugo and Longworth, 1966; Denton, 1991; Gilbert and Moore, 2005). Additionally, an apparent ability to inhibit membrane-bound and soluble ATPase was detected in *E. faecalis* (Figure 1-5; Harold et al., 1969).

Similarly to QACs, CHX is not completely removed through wastewater treatment, with low concentrations detected in the treated effluent (Matsushima and Sakurai, 1984; Ostman et al., 2017; Environment and Climate Change Canada [ECCC], 2019). Residues of CHX have also been described in the skin of patients (<4.69–600 mg/L) after CHX bathing, as well as in milk from cows treated with CHX teat dips and sprays (European Medicines Evaluation Agency [EMEA], 1996a; Popovich et al., 2012). CHX undergoes photodegradation but limited biodegradation and has a long half-life (e.g., 180–365 days in water, soil or sediment) (Kampf, 2018c; Environment and Climate Change Canada [ECCC], 2019). It tends to persist in water, suggesting potential for prolonged exposure far from the sources of discharge to the environment, and is “very toxic to aquatic life with long-lasting effects” according to ECHA (Kampf, 2018c; Environment and Climate Change Canada [ECCC], 2019; European Chemicals Agency [ECHA], 2023c). However, its bioavailability reduces over time by the adsorption of CHX to sediments and soil (Kampf, 2018c; Environment and Climate Change Canada [ECCC], 2019), potentially with a decreased impact over local microbiota.

PHMB is a polymeric biguanide, composed by 2 to 30 repeats of hexamethylene biguanide units, and possesses a bacteriostatic and bactericidal mechanism of action similar to the one described for CHX (Figure 1-6), although with a distinct initial interaction with the cytoplasmatic membrane (Davies et al., 1968; Ikeda et al., 1984; McDonnell and Russell, 1999; Gilbert and Moore, 2005; Kathuria et al., 2021). Given the polycationic nature of PHMB, the bridging occurs not only between pairs of adjacent phospholipids but rather there is the formation of a mosaic of single phospholipid type domains, each with different phase transition properties (Davies et al., 1968; Ikeda et al., 1984; McDonnell and Russell, 1999; Gilbert and Moore, 2005; Kathuria et al., 2021). An increased PHMB activity has been linked to higher levels of oligomerization in the Gram-negative *E. coli* (Broxton et al., 1983). PHMB has been used predominantly in concentrations between 30 and 32,000 mg/L in recreational water (e.g., swimming pools, artificial fountains) treatment, as well as in wound and burn antisepsis, surfaces and instrument disinfection in hospitals, dentists, farms and food handling settings, and in contact lens solutions, personal care products and fabric softeners preservation (Hübner and Kramer, 2010; Kampf, 2018d; Kathuria et al., 2021; Fox et al., 2022). PHMB shows very low human toxicity or risk of adverse effects (Gilbert and Moore, 2005; Grytsai et al., 2021; Fox et al., 2022; Rippon et al., 2023).

PHMB is very persistent in water, also showing a long half-life in this context, which may constitute an issue in aquatic environments as it is classified by ECHA, like CHX and QACs, as “very toxic to aquatic life with long lasting effects” (European Chemicals Agency [ECHA], 2017, 2023d; Kampf, 2018d). Also, it is considered to be hydrolytically and photolytically stable (European Chemicals Agency [ECHA], 2017). On the other hand, PHMB binds immediately to soils, except for sandy soil, and it is likely susceptible to some extent of biodegradation, although it is regarded as “non readily biodegradable” (O’Malley et al., 2006; Lucas, 2012; European Chemicals Agency [ECHA], 2017; Kampf, 2018d).

## 3 *In Vitro* testing of *Enterococcus* spp. susceptibility to cationic biocides

The *in vitro* assessment of *Enterococcus* spp. susceptibility to CBs has been performed using diverse methodologies, each selected to provide specific data relevant to the purpose of the test. The bactericidal efficacy claim of disinfectant or antiseptic products in Europe and the USA is supported by quantitative or qualitative tests simulating practical conditions specified in different standards according to their intended use (EPA - United States Environmental Protection Agency, 2012, 2018; Official Methods of Analysis of the AOAC International, 2013; CEN-CENELEC - European Committee for Standardization - European Committee for Electrotechnical Standardization, 2018). These may include different *Enterococcus* spp. reference strains as test organisms (EPA - United States Environmental Protection Agency, 2012; CEN-CENELEC - European Committee for Standardization - European Committee for Electrotechnical Standardization, 2018). However, such standardized tests required for assessing biocidal product efficacy may not be ideal for examining the susceptibility of *Enterococcus* spp. strains from diverse genomic and epidemiological backgrounds. They may also lack insights into long-term adaptation to biocide exposure within subinhibitory ranges, potentially not detecting evolving populations that remain susceptible to biocidal products. Thus, research studies assessing the susceptibility to CBs of *Enterococcus* spp. from diverse epidemiological and genomic backgrounds over the years have primarily relied on the *in vitro* determination of minimum inhibitory concentrations (MICs), mainly due to the methodology’s ease of use (Gnanadhas et al., 2013; Fox et al., 2022).

MIC is defined as the lowest antimicrobial concentration that inhibits the growth of the microorganisms, and it is usually measured in doubling dilutions (Clinical and Laboratory Standards Institute [CLSI], 1999, 2018; Gnanadhas et al., 2013; Fox et al., 2022). Additionally, some studies also include the determination of the minimal bactericidal concentrations (MBCs), corresponding to the minimum concentration that kills >99.9% of cells, which constitute a more suitable measure of susceptibility for most biocidal applications where the desired effect is to kill the microorganisms (Clinical and Laboratory Standards Institute [CLSI], 1999; Gnanadhas et al., 2013; Fox et al., 2022; Maillard and Pascoe, 2024).

Supplementary Table 1 shows several published MICs and MBCs of QACs (BC, DDAC, CE, CPC) and biguanides (CHX and PHMB), pointing to a good antimicrobial activity of these CBs against *Enterococcus* spp. isolates, when MICs or MBCs are compared to typical in-use concentrations. However, despite offering information on epidemiological variability and the monitoring of susceptibility trends within this genus, MICs or MBCs may not directly correlate with the bactericidal efficacy evaluated by the standards for disinfectant or antiseptic products approval (Kampf, 2022). This may occur even if the concentrations used correspond to those in the biocidal products because, in most susceptibility studies, MICs and MBCs are determined for unformulated CBs in simple aqueous solutions and biocidal products generally include other compounds that enhance CBs’ activity or stability (Cowley et al., 2015; Fox et al., 2022; Maillard and Pascoe, 2024). Some studies with biocidal formulations against *Enterococcus* spp. are available, showing MIC and MBC values higher or within the ranges of those in Supplementary Table 1 for unformulated CBs (McBain Andrew et al., 2004; Moore et al., 2008; Cowley et al., 2015; Günther et al., 2015; Bhardwaj et al., 2016; Ulusoy et al., 2016; López-Rojas et al., 2017; Piątkowska et al., 2021). However, these were evaluated at the endpoint of the MIC determination protocols used, corresponding typically to 24h or sometimes longer (48h), instead of the usually recommended disinfectant contact times in different contexts of 3 to 10 min (Maillard, 2005; Hong et al., 2017; Rutala et al., 2019). Additionally, the use of MIC and MBC may fail to detect tolerant persister subpopulations that are able to survive transient exposures to lethal biocide concentrations, potentially facilitating the evolution toward resistance, as observed for *E. coli* exposed to BC (Nordholt et al., 2021). Hence, tests to assess the efficacy of high concentrations of CBs, included or not in biocidal products, considering real exposure times are still lacking against *Enterococcus* spp. from diverse epidemiological and genomic backgrounds.

Most studies use the microdilution broth protocol described by the CLSI guidelines for antibiotic susceptibility testing (Clinical and Laboratory Standards Institute [CLSI], 1999, 2018) which has the advantage of allowing the comparison of data between different studies. However, it was designed primarily to assess the therapeutical success of antibiotics for infections’ treatment, which is reflected in the bacterial growth conditions specified (37°C; pH = 7.3) (Wales and Davies, 2015; Clinical and Laboratory Standards Institute [CLSI], 2018; Maillard and Pascoe, 2024). Thus, several other factors (e.g., variable temperature or pH, oxygen limitation, starvation, lower or higher bacterial density, bacterial growth phase) that can affect the efficiency of biocides in real application contexts, namely by altering the cytoplasmic membrane or reducing the cell’s metabolic activity, are not pondered (Zhang and Rock, 2008; Saito et al., 2014; Ran et al., 2015; Wiegand et al., 2015; Yoon et al., 2015; Gaca and Lemos, 2019; Fox et al., 2022; Maillard and Pascoe, 2024). Recently, we learned that *E. faecium* and *E. faecalis* isolates from different epidemiological and clonal backgrounds exhibited decreased susceptibility (MIC and MBC increases of two to eightfold) to BC at 22°C and/or pH = 5, compared to standard conditions (37°C; pH = 7.3) (Pereira et al., 2023), confirming the influence of external growth conditions on CBs susceptibility.

Furthermore, *Enterococcus* are also regularly found within multicellular communities such as biofilms, both on wet environments and dry surfaces, in various settings (Ch’ng et al., 2019). Biofilms are usually associated with a decreased susceptibility to biocides via several mechanisms such as persister cells and surrounding extracellular polymeric matrix that forms a barrier to the diffusion of biocides through the biofilm (Ch’ng et al., 2019; Wicaksono et al., 2021; Maillard and Pascoe, 2024). Although a good biocidal activity remains generally described against *Enterococcus* spp. biofilms (Lima et al., 2001; Arias-Moliz et al., 2010; Baca et al., 2011; Ravi Chandra et al., 2015; Komiyama et al., 2016; Valverde et al., 2017; Machuca et al., 2019; Günther et al., 2021), one study reported a decreased susceptibility in enterococcal biofilms compared to planktonic cells, for BC, DDAC, CHX and PHMB, of around twofold between minimum biofilm eradication concentrations (MBECs) and MBCs (Cowley et al., 2015). Also, in a recent study, a BC concentration of 80 mg/L was not sufficient to eradicate *E. faecium* and *E. faecalis* biofilms for which respective planktonic cells’ MBCs varied from 10 to 40 mg/L (Salamandane et al., 2023).

Finally, given the absence of established standardized protocols for the study of biocide susceptibility of different bacterial strains or collections, prudence is necessary when comparing MIC/MBC data across different studies, making comprehensive epidemiological analysis difficult (Buffet-Bataillon et al., 2012; Maillard and Pascoe, 2024). A clear example of such issue is the broad range of CHX MICs available for the *E. faecalis* ATCC 29212, from <1 to 27 mg/L (Supplementary Table 1), determined using different methodologies. Factors such as the incubation time, bacterial growth phase (e.g., exponential *vs* stationary), growth culture media or the type of plate plastic can influence the susceptibility to CBs *in vitro*, conducting to diverse outcomes for the same strains (Clinical and Laboratory Standards Institute [CLSI], 1999; Bock et al., 2018).

Despite the limitations of data analysis related to the inconsistency of the methods used and the potential implications on the applicability and relevance of *in vitro* data in real environments, the diverse susceptibility testing methodologies (e.g., altered growth conditions, planktonic cells or biofilms, biocidal formulations *vs* unformulated CBs) may be valuable in different contexts, provided they are standardized. Meanwhile, more studies using biocidal formulations, along with the inclusion of biofilms or modified growth conditions simulating real scenarios, are crucial to ascertain the activity of CBs against *Enterococcus* populations and to conclude about the appropriate course of action.

Besides susceptibility assessment methodologies, another aspect needing standardization, also linked to susceptibility testing, is the terminology used to define the decreased susceptibility of bacteria to biocides (Maillard and Pascoe, 2024). When the increased MICs or MBCs do not reach in-use concentrations of a biocide, it has been described using the terms “tolerance” or “decreased susceptibility” (Maillard, 2007; Rutala et al., 2019; Wand and Sutton, 2022; Boyce, 2023). As the term “tolerance” has been used with different meanings to characterize bacterial susceptibility to biocides or antibiotics (Brauner et al., 2016), for the purpose of this review we will use the term “decreased susceptibility”. On the contrary, if the decreased susceptibility implies that the microorganisms are not inactivated by the in-use concentrations of a biocide, then the term “resistance” to the biocide is applied (Maillard, 2007; Rutala et al., 2019; Wand and Sutton, 2022; Boyce, 2023).

## 4 Susceptibility to cationic biocides of *Enterococcus* spp. from diverse sources and time frames

More and more the wide use of antiseptics and disinfectants in particular environments (e.g., hospitals, the food chain) has been a cause of concern given the possibility that repeated exposure to subinhibitory concentrations of these agents may progressively select for populations with decreased susceptibility to these antimicrobials (Fraise, 2002; Meyer and Cookson, 2010; Kampf, 2018e; Maillard and Pascoe, 2024). As a baseline to monitor the susceptibility evolution trends to CBs over the years or among strains from diverse sources under a gradient of selective subinhibitory pressures, setting epidemiological cut-off (ECOFF) values could be a useful tool (Turnidge et al., 2006; Morrissey et al., 2014; Kahlmeter and Turnidge, 2022). ECOFFs are established based on the MIC or MBC distributions of an antimicrobial for each bacterial species, and correspond to the minimum concentration above which bacterial strains have phenotypically detectable acquired reduced susceptibility mechanisms (Turnidge et al., 2006; Morrissey et al., 2014; Kahlmeter and Turnidge, 2022). Although the methods used to determine MICs or MBCs may not accurately reflect resistance to biocides under real-world application conditions, as previously discussed, the purpose of setting ECOFF values is not to separate between resistant or susceptible isolates to biocide products, but rather to distinguish non-wild-type (those with acquired reduced susceptibility mechanisms) from wild-type strains (Turnidge et al., 2006; Morrissey et al., 2014; Kahlmeter and Turnidge, 2022).

Few individual analyses have proposed CBs’ ECOFF values based on their *Enterococcus* spp. collection’s MIC and MBC distributions, including isolates from different sources, geographical regions and time frames (Morrissey et al., 2014; Duarte et al., 2019; Kheljan et al., 2022; Pereira et al., 2022). For CHX, MIC ECOFFs of 8 mg/L and 32 mg/L and an MBC ECOFF of 64 mg/L have been proposed for *E. faecium*, whereas, for *E. faecalis*, MIC ECOFFs of 8 mg/L, 16 mg/L and 64 mg/L and MBC ECOFFs of 64 mg/L or higher have been recommended (Morrissey et al., 2014; Kheljan et al., 2022; Pereira et al., 2022). For BC, MIC ECOFFs of 8 mg/L and 16 mg/L and an MBC ECOFF of 16 mg/L have been estimated for both *E. faecium* and *E. faecalis* (Morrissey et al., 2014; Kheljan et al., 2022). However, the variation in ECOFFs proposed by different studies underscores the need for comprehensive analyses of MIC and MBC distributions across the various species-biocide pairs. These must be conducted using diverse *Enterococcus* spp. collections and laboratories to address potential biological, methodological, and interlaboratory variations. Such an approach aligns with EUCAST recommendations for antibiotics and is crucial for establishing definitive ECOFFs for biocides (Kahlmeter and Turnidge, 2022).

In one of the studies proposing ECOFFs to CHX for *E. faecalis*, even though the whole population was considered wild type by the statistical model recommended for the ECOFF estimation, differences in the susceptibility were detected among isolates from diverse sources and years (Pereira et al., 2022). Similarly, other authors have found significant differences across their *Enterococcus* spp. collections (Schwaiger et al., 2014; Duarte et al., 2019; Sobhanipoor et al., 2021; Kheljan et al., 2022; Pereira et al., 2022, 2023). Most found a higher occurrence of strains with decreased susceptibility to CHX, DDAC, or BC among clinical isolates comparing to *Enterococcus* from other origins included in the same study (Schwaiger et al., 2014; Guzman Prieto et al., 2017; Duarte et al., 2019; Sobhanipoor et al., 2021; Pereira et al., 2023), although contradictory data has also been reported for BC (Sobhanipoor et al., 2021; Kheljan et al., 2022). Moreover, an increase in the mean CHX MICs and MBCs of human infection *E. faecalis* isolates over the years, between 2001 and 2020, has been recently reported (Pereira et al., 2022). Beyond the clinical environment, a significant increasing trend in the BC MICs over time has also been detected in *E. faecium* isolated from the food chain, including food-animal production settings, meat of animal origin, and other food products (Pereira et al., 2023). Furthermore, within populations of *E. faecalis* and *E. faecium* from these settings, higher average CHX MICs and MBCs have been identified compared to other sources (Kheljan et al., 2022; Pereira et al., 2022). Of note, most of these studies did not find any particular genotype justifying the evolution of phenotypes between sources or time frames (Schwaiger et al., 2014; Kheljan et al., 2022; Pereira et al., 2022, 2023).

Available data suggest an adaptation of *Enterococcus* populations in settings where they are exposed to CBs. Of note, an increase in the CHX MIC_50_ (minimum concentration that inhibits the growth of 50% of the isolates), from 2 to 8 mg/L, and MIC_90_ (minimum concentration that inhibits the growth of 90% of the isolates), from 16 to 32 mg/L, was detected in vancomycin-resistant *E. faecium* recovered from patients’ infections or colonization after daily CHX bathing was instituted in the hospital ward, compared to isolates recovered before the intervention, suggesting that prolonged exposure to this biocide might indeed select for decreased susceptibility (Mendes et al., 2016). Nonetheless, large longitudinal metadata analyses of the populations’ dynamics in such contexts are critically needed for more supported conclusions. Currently, most available CBs susceptibility studies lack objective data on local biocide consumption (e.g., type of compound, amount used, compliance with effective biocide application practices, “during use” concentrations) or on the occurrence of subinhibitory concentrations (Maillard and Pascoe, 2024). This hinders the establishment of a clear cause-and-effect relationship of biocide use and *Enterococcus* spp. evolved phenotypes. Most studies also use a limited number of *Enterococcus* strains, lack clonal or genotypic characterization, and show a low source diversity, making it challenging to have a global perspective on the evolution of susceptibility to CBs in particular *Enterococcus* spp. populations or environments.

## 5 Evolution of *Enterococcus* spp. serially exposed to subinhibitory concentrations of cationic biocides *In Vitro*

Despite unclear cause-and-effect relationships in the previously mentioned studies that detected CBs phenotypic evolutions among field *Enterococcus* spp. isolates from diverse sources or dates, the hypothesis that exposure to diverse subinhibitory concentration gradients of CBs could lead to decreased susceptibility has been tested *in vitro* (Cowley et al., 2015; Tezel and Pavlostathis, 2015). These tests, in which diverse *Enterococcus* spp. are exposed to low CBs concentrations similar to those found in different environments, such as residual concentrations in treated surfaces (e.g., skin, food products, abiotic surfaces) or in surface or residual waters as a result of indirect contamination, contribute to identifying the cellular mechanisms involved in *Enterococcus* spp. response or adaptation to CBs as well as to other antimicrobials, including antibiotics (Cowley et al., 2015; Tezel and Pavlostathis, 2015).

*Enterococcus* spp. passages with BC, DDAC, CPC, CE, CHX, and PHMB have resulted in MIC increases of 1.2 to more than 100-fold, that were strain-specific within each species (Supplementary Table 2). In most cases, MICs and MBCs of *Enterococcus* spp. adapted strains remained below the in-use concentrations of CBs. However, for one *E. casseliflavus* and one *E. faecalis* treated with increasing CE and PHMB concentrations, respectively, MICs and, for the *E. faecalis*, MBCs reached the in-use concentrations range and were stable or only partially reversed after several biocide-free subcultures (Supplementary Table 2) (European Medicines Evaluation Agency [EMEA], 1996b; Cowley et al., 2015; Gadea et al., 2017b; Fox et al., 2022). Also, for most CHX experiments, including different species, MICs reached the concentrations typically used for preservation (25–100 mg/L) after exposure (Supplementary Table 2; Maillard, 2005; Kampf, 2018c; Fox et al., 2022).

The decreased bacterial susceptibility following the adaptation protocols may be explained by several factors such as changes in membrane fatty acid composition, differential expression or mutations in efflux pumps, induction of stress responses, among others (Cowley et al., 2015). Although some of the analyses revealed stable phenotypes, suggesting genotypic adaptation rather than noninheritable physiological or metabolic adaptation mechanisms (Baquero and Coque, 2014), these were scarcely investigated. Bhardwaj et al. (2017) identified significant changes in membrane phospholipids and mutations in several genes with previously predicted or experimentally confirmed roles in decreased susceptibility to CHX, among CHX-adapted *E. faecium* strains. In particular, all of them shared a mutation (A290V) in the gene *efrE*, which encodes one subunit of the heterodimeric ATP-binding cassette (ABC) transporter EfrEF whose deletion resulted in increased CHX susceptibility (Bhardwaj et al., 2017). Additionally, increased surface hydrophobicity was detected in *E. faecalis* passaged in the presence of CPC as well as in those serially exposed to CHX for which a change in the protein profile was also found (Kitagawa et al., 2016). Findings such as these suggest the occurrence of complex bacterial adaptation mechanisms to CBs and underscore the importance of more in-depth analyses employing advanced technologies, like whole-genome sequencing (WGS) and transcriptomics, to identify possible drivers of *Enterococcus* spp. decreased susceptibility.

The effects of *in vitro* serial exposure to subinhibitory concentrations of CBs in *Enterococcus* spp. were not limited to the decreased susceptibility to that specific biocide. Considerable increases of several fold in the MICs of other biocides (2 to >100 fold), reaching in-use concentrations in many cases, have also been detected (Supplementary Table 2; Bhardwaj et al., 2017; Gadea et al., 2017a,b). On the other hand, an increase in susceptibility to CE occurred in CPC-adapted *Enterococcus* spp. and BC-adapted *E. faecium, E. faecalis*, and *Enterococcus* spp., to BC in CPC-adapted *Enterococcus* spp., to didecyldimethylammonium bromide in CPC-adapted *Enterococcus* spp., to CPC in BC-adapted *Enterococcus* spp. and CHX-adapted *Enterococcus* spp., and to triclosan in CHX-adapted *E. casseliflavus* and CPC-adapted *Enterococcus* spp. (Gadea et al., 2017a,b).

All in all, despite the *in vitro* research suggesting that bacteria can adapt to CBs exposure, evidence of such rapid adaptation in the environment is scarce (Cowley et al., 2015; Maillard and Pascoe, 2024). Multiple external factors specific of each setting (e.g., physicochemical, wide range of CBs concentrations, presence of additional compounds with antimicrobial properties, presence of organic matter), the different physiological state of bacterial populations (e.g., changes in metabolic activity and gene expression), among others, may not allow for the conditions required for *Enterococcus* bacteria to adapt (Fox et al., 2022; Maillard and Pascoe, 2024). Also, another hypothesis that might explain the limited correlation data between biocide use and an evolution toward decreased susceptibility in real environmental contexts are the loss of the adaptation mechanism or the struggle of adapted populations to compete in their microbial communities when the CB stress is removed (Cowley et al., 2015). This once again supports that field studies are a critical research gap, as mentioned throughout this review, namely longitudinal analyses with appropriate controls and with collection of data on the biocide concentrations used, exposure times and time intervals between exposures, simultaneous application of other compounds, among others.

## 6 Genotypes of decreased susceptibility to cationic biocides among *Enterococcus* spp.

CBs’ mechanism of action is complex and comprises multiple targets, including the cytoplasmic membrane as well as intracellular components such as proteins and nucleic acids, in a concentration-dependent manner, as previously discussed (Salton, 1951; Hugo and Longworth, 1964, 1966; Harold et al., 1969; Denton, 1991; Gilbert and Moore, 2005; Maillard and Pascoe, 2024). Decreased susceptibility to CBs in Gram-positive bacteria has been mainly attributed to efflux pumps (Tezel and Pavlostathis, 2015; Fox et al., 2022), which may prevent or reduce CB’s antibacterial action by exporting them from the cytoplasm or the cytoplasmic membrane, up to a certain CB concentration (Putman et al., 2000; Boyce, 2023). *Enterococcus* spp. have been found to harbor several acquired genes encoding efflux pumps located on the cytoplasmic membrane, including the well-known *qac, bcrABC* and *oqxAB* genes, all demonstrated to be implicated in the decreased susceptibility to QACs by functional studies in Gram-positive or Gram-negative bacteria, and, in the case of some *qac* genes, also to CHX. Intrinsic heterodimeric ABC transporters, like EfrEF in *E. faecium* and *E. faecalis*, and mutations in regulatory genes, such as the DNA-binding response regulator (ChtR), have also been shown to impact CHX susceptibility.

However, as will be detailed throughout this chapter, for most genotypes of decreased susceptibility to CBs, their role in *Enterococcus* spp. antimicrobial susceptibility is hypothesized based on functional assays in other bacterial genus and/or epidemiological studies in *Enterococcus* spp. in which genotypes and phenotypes are correlated without molecular support. Further characterization of the functionality of such genes, through, for example, gene deletion and complementation studies, is required to completely elucidate their role in *Enterococcus* spp. susceptibility to CBs. Nonetheless, the potential gene exchange with diverse phyla, both of Gram-positive and Gram-negative bacteria, in contexts where CBs are present in a wide range of concentrations, is noteworthy and needs further exploration as the same genotypes in diverse genetic or epidemiological backgrounds may be associated with diverse outcomes of CBs susceptibility.

### 6.1 *qac* genes

The *qac* genes detected in *Enterococcus* spp. are often plasmid-located and belong to two major classes of efflux pump systems: the major facilitator superfamily (MFS; e.g., *qacA/B*) and the small multidrug resistance family (SMR; e.g., *qacC, qacE, qacEΔ1, qacG, qacJ, qacZ*) (Ortega Morente et al., 2013; Rizzotti et al., 2016; Pereira et al., 2023). They are proton motive force-dependent efflux pumps integrated in the cytoplasmic membrane via transmembrane segments (Bay et al., 2008; Cervinkova et al., 2013; Ortega Morente et al., 2013; Wassenaar et al., 2015; LaBreck et al., 2020). Among *qac* genes, *qacZ* is the only one for which its role in decreased susceptibility to QACs has been demonstrated in *Enterococcus* spp., by complementation of an *E. faecalis* strain with this gene (Braga et al., 2011). For the remaining genes, such functional assays were performed either in *S. aureus*, for *qacA/B*, *qacC, qacG* or *qacJ*, or in *E. coli*, for *qacE* or *qacEΔ1*, showing their impact on decreased susceptibility to QACs or CHX (only for *qacA*) (Littlejohn et al., 1992; Paulsen et al., 1993; Heir et al., 1999; Bjorland et al., 2003).

QacA has been associated with decreased susceptibility to various cationic compounds including QACs, CHX, diamides, intercalating dyes, among others, in *S. aureus* (Littlejohn et al., 1992; Cervinkova et al., 2013; Wassenaar et al., 2015; LaBreck et al., 2020). It is encoded by the *qacA* gene which is closely related to *qacB*, with the encoded proteins differing at amino acid position 323 (Paulsen et al., 1996; Ortega Morente et al., 2013; Wassenaar et al., 2015). QacA features aspartic acid at this position, while QacB has alanine, impacting substrate recognition and binding and reducing QacB’s efflux activity of divalent cations (Littlejohn et al., 1992; Paulsen et al., 1996; Ortega Morente et al., 2013; Wassenaar et al., 2015). Both genes are regulated by a TetR/CamR transcriptional regulator, QacR, that binds to the *qacA/B* promoter, inhibiting its expression (Galluzzi et al., 2003; Wassenaar et al., 2015; LaBreck et al., 2020). When substrates of QacA/B directly bind to QacR, the regulator dissociates from the promoter and allows for expression of the efflux pump genes (Galluzzi et al., 2003; Wassenaar et al., 2015; LaBreck et al., 2020).

Previous studies have reported different occurrence rates of *qacA/B* in collections of *E. faecalis* and *E. faecium* isolates with diverse epidemiological backgrounds (Supplementary Table 3; Bischoff et al., 2012; Rizzotti et al., 2016; Sommer et al., 2019; Kheljan et al., 2022). These have shown a susceptible phenotype to DDAC, BC and CHX at concentrations much lower than those present in biocide-containing products. One of the *qacA/B*-carrying *E. faecalis*, recovered from human blood, had a higher DDAC MIC of 2.45–3.5 mg/L compared to isolates without this gene (MIC of 1.05 mg/L), whereas the other *qacA/B*-carrying *E. faecalis*, isolated from cattle, did not present an increased MIC value for DDAC (Supplementary Table 1; Bischoff et al., 2012). The authors suggested the different phenotypes could be linked to the nucleotide polymorphisms between *qacA/B* sequences in the two isolates (Bischoff et al., 2012), but additional analyses to confirm the role of such mutations in decreased susceptibility to DDAC are needed. In *E. faecium*, a decreased susceptibility to CHX (MIC of 14 mg/L) was detected in a swine production chain strain (EA26; *qacA/B*-positive) compared to MICs of 4–10 mg/L for *qacA/B* negative isolates (Supplementary Table 1; Rizzotti et al., 2016). On the other hand, the BC MIC of *E. faecium* EA26 was within the range determined for isolates without *qacA/B* in this study (2–4 mg/L) (Supplementary Table 1; Rizzotti et al., 2016). In a more recent study including 647 *Enterococcus* spp. isolates from different sources in Iran, BC and CHX MIC_90_ of *E. faecalis* and *E. faecium* harboring or not *qacA/B* were similar (Kheljan et al., 2022). Additionally, on publicly available genomes at the NCBI (until 28/4/2022), the gene *qacA/B* was also found among two *E. faecalis* and one of *E. faecium*, all from recent human infections in South Africa (Pereira et al., 2023).

The *qacC* gene, also known as *ebr*, *smr* (for staphylococcal multidrug resistance) or *qacD*, confers decreased susceptibility to QACs and β-lactam antibiotics in Gram-positive and Gram-negative bacteria, and is usually located on conjugative or small rolling-circle replicating (nonconjugative) plasmids (Lyon and Skurray, 1987; Littlejohn et al., 1990, 1992; Fuentes et al., 2005; Ortega Morente et al., 2013; Wassenaar et al., 2015; LaBreck et al., 2020). Its expression does not require a transcriptional regulator and the corresponding QacC protein is 107 amino acids long (Littlejohn et al., 1990; Wassenaar et al., 2015; LaBreck et al., 2020). QacC has been found in six *E. faecalis* isolated from pediatric bloodstream infections, human stool and cheese (Supplementary Table 3), as well as in 29 *E. faecalis* and seven *E. faecium* genomes available on NCBI (until 28/4/2022) (Bischoff et al., 2012; Sommer et al., 2019; Pereira et al., 2023). The susceptibility to DDAC was tested for the two non-clinical isolates and showed that both had an MIC of 1.05 mg/L similar to the *qacC* negative isolates (Supplementary Table 1; Bischoff et al., 2012).

The genes *qac*E and the partially deleted derivative known as *qac*EΔ*1*, resulting from the insertion of a DNA segment containing a sulfonamide resistance gene near the 3’ end of the *qacE* gene, are commonly found in integrons of a broad range of Gram-negative bacteria (Paulsen et al., 1993; Kazama et al., 1998a,b). They were associated with decreased susceptibility to QACs, with *qacE* associated with lower susceptibility levels than *qacEΔ1* (Paulsen et al., 1993). In *Enterococcus*, *qacE* was found among three vancomycin-resistant *E. faecium* from patients’ infections or colonization (Brazil, 2005–2009) (Supplementary Table 3) but an association with CHX MICs was not established (Mendes et al., 2016). Similarly, *qacEΔ1* has been detected in nine clinical *E. faecalis* isolates (Japan, 1996) and in 44 *E. faecalis* and 73 *E. faecium* from diverse sources in Iran (2018–2020) (Supplementary Tables 1, 3), that did not show increased BC and CHX MIC_90_ compared to *qacEΔ1* negative isolates (Kazama et al., 1998a; Kheljan et al., 2022).

The gene *qacG* has been detected in *E. faecium* 8D1-48 (NCBI; until 28/4/2022), a soil isolate, and *qacJ* in two *E. faecalis* strains from human infections (NCBI; until 28/4/2022) and cattle processed meat (Supplementary Table 3; Matle et al., 2023; Pereira et al., 2023). QacG and QacJ have a high protein sequence identity between them (82.6%) and with QacC (>69%), belonging to the SMR family (Heir et al., 1999; Bjorland et al., 2003; Wassenaar et al., 2015). No phenotypic assays are available to infer about their role in *Enterococcus* spp. decreased susceptibility to QACs.

The *qacZ* and *qacH*, identified in *Enterococcus* spp. and *Staphylococcus* spp., respectively, share a high sequence similarity (98% nucleotide identity) but different substrates (Braga et al., 2011; Silveira et al., 2015). The role of gene *qacZ* in *Enterococcus* spp. decreased susceptibility to QACs has been shown, but not to ethidium bromide or proflavine, which are also substrates of the efflux pump coded by *qacH* (Braga et al., 2011; Silveira et al., 2015). Braga et al. (2011) found a high occurrence of *qacZ* among different *Enterococcus* spp. isolated from Portuguese clinical settings (63%) and dairy products (70%), although a correlation between the prevalence of the gene and BC or CHX MICs was not detected (Supplementary Tables 1, 3). In a different collection, only one ST17 *E. faecium* (E241), recovered from hospital sewage in 2002, also in Portugal, carried *qacZ* (Supplementary Table 3). Similarly, its BC and CHX MIC and MBC remained low and within the ranges also observed for isolates without such gene (Supplementary Tables 1, 3; Silveira et al., 2015; Duarte et al., 2019; Pereira et al., 2023). Twenty *E. faecalis* and 12 *E. faecium qacZ*-carrying genomes available on NCBI (until 28/4/2022), mostly from human infections in different countries and years (1987–2014), have also been reported (Pereira et al., 2023).

Generally, similar phenotypes to CBs have been described for *Enterococcus* with or without *qac* genes, suggesting that their activity may not have an impactful outcome in the susceptibility to CBs, that other mechanisms such as the presence or differential regulation of other efflux pumps may also influence the resulting phenotypes, or that the necessary conditions for the expression of decreased susceptibility genotypes are not satisfied by the methodologies used (Braga et al., 2011; Bischoff et al., 2012; Duarte et al., 2019; Kheljan et al., 2022; Pereira et al., 2023). Also, with few exceptions (Kazama et al., 1998a; Braga et al., 2011; Sommer et al., 2019; Kheljan et al., 2022), a low occurrence of *qac* genes among *Enterococcus* spp. isolates and genomes (available on NCBI) has been reported (Bischoff et al., 2012; Schwaiger et al., 2014; Silveira et al., 2015; Rizzotti et al., 2016; Ignak et al., 2017; Duarte et al., 2019; Sobhanipoor et al., 2021; Pereira et al., 2023). These data suggest that *qac* genes may not have a significant impact on the response of *Enterococcus* spp. to CBs exposure, although such conclusion may be biased by the few published studies, by the *Enterococcus* collections included in such analyses or the public genomes available. Of note is the detection of *qac* genes on transferable plasmids, potentially facilitating their transmission, via horizontal gene transfer, within the microbial communities and settings *Enterococcus* spp. are part of (Silveira et al., 2015; Wassenaar et al., 2015; LaBreck et al., 2020; Pereira et al., 2023). Indeed, the sequences of QacA/B, QacC, QacG, QacJ and QacZ, which were predominantly identified in human *Enterococcus*, were mainly shared with *Staphylococcus* isolates associated with human colonization and infection, in which they have been primarily and mostly described (Wassenaar et al., 2015; Pereira et al., 2023).

### 6.2 *qrg*

The *qrg* gene belongs to the SMR family, as most *qac* genes, and has been shown to encode a fourfold decreased susceptibility to cetyltrimethylammonium bromide, by deletion and complementation assays, in one *Streptococcus oralis* isolate from the human oral cavity, in which the gene was first described (Ciric et al., 2011). Among *Enterococcus*, *qrg* was harbored only by one ST6 *E. faecalis* DVT_1043 (available on NCBI; until 28/4/2022), a human infection strain isolated in the USA in 2020 (Pereira et al., 2023). *Streptococcus* spp. was the predominant genus (98%, *n* = 183/187) sharing an identical Qrg sequence with the DVT_1043 strain, among the five genera (14 species) in which it was found (Pereira et al., 2023). CBs susceptibility studies on *qrg*-carrying *Enterococcus* have not yet been published, with the impact of this gene in *Enterococcus* susceptibility to QACs still to explore.

### 6.3 *bcrABC* cassette

The efflux system encoded by the *bcrABC* cassette is associated with decreased susceptibility to QACs and it is predominantly harbored by plasmids of *Listeria* spp., although a chromosomal location is also possible (Elhanafi et al., 2010; Katharios-Lanwermeyer et al., 2012; Dutta et al., 2013; Jiang et al., 2016). It is composed by a putative transcriptional regulator of the TetR family, *bcrA*, and two SMR genes, *bcrB* and *bcrC* (Elhanafi et al., 2010; Dutta et al., 2013).

Although, in a previous study, *bcrABC* genes were not detected among a collection of over 200 *E. faecium* and *E. faecalis* from human, animal, food and aquatic sources from eight countries and spanning 25 years (Supplementary Table 3), these were the most prevalent genes encoding decreased susceptibility to CBs among *Enterococcus* spp. genomes available at the NCBI database (*n* = 22,428; until 28/04/2022), when compared to *qacA/B*, *qacC, qacG, qacJ, qacZ, qrg, bcrABC*, and the *oqxAB* genes (Pereira et al., 2023). They were more frequent in *E. faecalis* than in *E. faecium* or *E. lactis*, as well as in the food chain compared to other sources, probably as a reflection of the microbial communities and settings in which these genes circulate (Pereira et al., 2023). Indeed, an identical *bcrABC* gene cluster to that found in *Enterococcus* was mostly detected in the food pathogen *Listeria monocytogenes* (97% among the 10 species, corresponding to six different genera, in which it was identified) (Pereira et al., 2023). While the decreased susceptibility to CBs has been confirmed for *Listeria monocytogenes* by transfer of the *bcrABC* genes to a plasmid-cured strain (Elhanafi et al., 2010), functional studies in *Enterococcus* to support their role in this different host are needed.

### 6.4 *oqxAB* genes

The multidrug efflux pump encoded by the *oqxAB* cassette belongs to the resistance nodulation division family (RND) and has been most commonly found on the chromosome and/or conjugative plasmids of *Enterobacterales* (Li et al., 2019). In *E. coli* harboring a plasmid with or without the *oqxAB* genes, their functionality has been shown to decrease the susceptibility to the CBs BC, CE and CHX, as well as to multiple antibiotics (Hansen et al., 2007). However, in *Enterococcus* spp., only their role in antibiotic susceptibility has been demonstrated so far (Yuan et al., 2018).

Among the rare studies in which *Enterococcus* isolates were screened for the presence of *oqxAB* (Supplementary Table 3), high percentages of strains harboring these efflux pump genes were detected in manure of food-producing animals in China (*oqxA:* 79%; *oqxB:* 66%), which may be due to the extensive use of quinoxalines in animal husbandry in this country as suggested by the authors (Yuan et al., 2018). However, similarly to *qrg* or *bcrABC*, the susceptibility to CBs of any *oqxAB*-positive *Enterococcus* isolates has not yet been determined. The *oqxAB* genes seem more prevalent in *E. faecalis* than in *E. faecium*, and filter-mating experiments showed their transferability between *E. faecalis* strains (Yuan et al., 2018). The OqxAB variant found in *Enterococcus* has been identified in *E. coli* and *Salmonella enterica* available at the NCBI database (until 28/04/2022), recovered predominantly from the food chain (Pereira et al., 2023).

### 6.5 *emeA*

EmeA is an enterococcal multidrug-resistant efflux pump, homolog to *S. aureus* NorA (32% identity), that belongs to the major facilitator superfamily (MFS) and has been associated with decreased susceptibility to QACs, dyes and different antibiotics, in *E. faecalis* and *E. coli* complemented with this gene (Jonas et al., 2001; Lee et al., 2003a). Several studies screening the presence of the gene *emeA* among *Enterococcus* spp. isolates have been published (Supplementary Table 3). In *E. faecalis* it is considered to contribute to intrinsic drug resistance, being identified in all complete and annotated *E. faecalis* genomes from GenBank on September 1^st^ of 2020 (Panthee et al., 2021). However, in the few epidemiological analysis where its impact on susceptibility to CBs has been assessed, no decreased susceptibility was observed for *emeA* positive *Enterococcus* spp. compared to isolates without such gene (Rizzotti et al., 2016; Kheljan et al., 2022), except in one case in which the presence of this gene was significantly associated with decreased susceptibility to CHX but not to BC (Sobhanipoor et al., 2021).

### 6.6 *efrAB*

EfrAB is an *Enterococcus* heterodimeric ABC multidrug efflux pump, chromosomally encoded by the *efrA* and *efrB* genes, that transports multiple dyes and antibiotics, including fluoroquinolones, in *E. faecalis* (Davis et al., 2001; Lee et al., 2003b; Lubelski et al., 2007; Hürlimann et al., 2016). Although a suspected role for *efrAB* in *Enterococcus* spp. susceptibility to CBs has been suggested (Lavilla Lerma et al., 2014; Sobhanipoor et al., 2021), functional gene studies are still lacking to confirm it.

EfrAB has been reported in *Enterococcus* spp. in different percentages (Supplementary Table 3), which may be related to a low sensitivity of detection methods to identify potential gene variability that remains to be assessed. On the other hand, it has been consistently found in a high occurrence (50 – 100%) in *E. faecalis* collections (Supplementary Table 3). Lavilla Lerma et al. (2014) described that all the *E. faecalis* with decreased susceptibility to CHX included in their study harbored *efrAB*, although, in *E. faecium* strains with decreased susceptibility, only 12% carried such genes. Recently, also Sobhanipoor et al. (2021) revealed a significant association between the presence of these genes and *Enterococcus* decreased susceptibility to CHX, but not to BC, similar to what was observed for *emeA*.

### 6.7 *chlR-efrEF*

EfrEF is another intrinsic heterodimeric multidrug ABC transporter, encoded by the genes *efrE* and *efrF*, with a drug efflux profile similar to that of EfrAB (Hürlimann et al., 2016; Li and Palmer, 2018). Furthermore, EfrEF’s role in decreased susceptibility to CHX has been shown both in *E. faecium* and *E. faecalis*, by deletion and complementation experiments (Bhardwaj et al., 2017; Li and Palmer, 2018). Using transcriptomic analysis to identify the genes upregulated in *E. faecalis* V583 under CHX exposure, Li and Palmer (2018) detected *efrEF* to be the most highly upregulated genes. Their overexpression was mediated by ChlR, a putative MerR family transcription regulator, encoded by *chlR* located upstream of *efrEF* (Li and Palmer, 2018). Deletion of the *chlR* gene resulted in increased susceptibility to CHX and was decreased in the complemented strain, as for *efrE* or *efrF* (Li and Palmer, 2018).

Following the observations made in these previous studies with a restricted set of strains, a large collection of 666 *E. faecalis* genomes from diverse epidemiological and clonal backgrounds was screened for the occurrence and variability of the *chlR*-*efrEF* genes (Pereira et al., 2022). The *efrEF* operon was detected in all but one isolate (Supplementary Table 3), with 5% carrying genes coding for incomplete ChlR, EfrE or EfrF proteins (Pereira et al., 2022). Most of these corresponded to EfrE-truncated *E. faecalis* identified as ST40 and were predominantly recovered from humans (Pereira et al., 2022). Isolates harboring incomplete ChlR-EfrEF had consistently low MICs ( ≤ 1mg/L, with rare exceptions) contrasting with those with complete operons (MIC = 2–8 mg/L, with 2 exceptions), whereas MBCs remained similar to those of non-truncated *E. faecalis* (Pereira et al., 2022). A broad range of mutations was identified among the isolates with complete ChlR-EfrEF proteins, but no correlation between specific mutations and CHX susceptibility was recognized (Pereira et al., 2022).

Similarly, *efrE* and *efrF E. faecium* orthologs were also upregulated in *E. faecium* 1,231,410 in response to CHX exposure (Bhardwaj et al., 2016). Deletion of the *efrEF* operon rendered this strain more susceptible to the biocide, whereas complementation restored the CHX phenotype (Bhardwaj et al., 2017). Moreover, *in vitro* serial exposure of *E. faecium* 1,231,410 to subinhibitory concentrations of CHX selected for a mutation in *efrE* (A290V) that was shown to confer decreased susceptibility to CHX (Bhardwaj et al., 2017). Several ChlR-EfrEF and promoter mutations were detected among 33 *E. faecium* and 4 *E. lactis* (former *E. faecium* Clade B) isolates from various sources and years (Supplementary Table 3), but not the A290V associated with decreased susceptibility to CHX, which was also absent in 980 *E. faecium* genomes from field isolates coding for a complete EfrE protein published in the GenBank database in December 2018 (Duarte et al., 2019).

### 6.8 *chtRS*

The conserved DNA-binding response regulator ChtR along with histidine kinase sensor ChtS form a putative two-component regulatory system (2CS) that has been demonstrated to be implicated in decreased susceptibility to CHX in *E. faecium*, through deletion and complementation experiments (Guzman Prieto et al., 2017; Duarte et al., 2019). Additionally, strains with deleted *chtR* and *chtS* showed a compromised growth and morphology under CHX exposure that was reverted when the mutations were complemented in trans (Guzman Prieto et al., 2017). Furthermore, similar assays revealed that a nonsynonymous single nucleotide polymorphism in *chtR*, leading to an amino acid substitution (P102H), predominantly found in clinical isolates, was linked to a CHX decreased susceptibility phenotype (Guzman Prieto et al., 2017; Duarte et al., 2019). The P102H mutation is located in the dimerization interface of the signal receiver domain of ChtR, which might affect the activation and function of the response regulator (Guzman Prieto et al., 2017). The genes *chtR* and *chtS* are predicted to be part of an operon, named 2CS-CHX^T^, also composed by genes related to sugar and amino acid transport (Duarte et al., 2019). Several other 2CS-CHX^T^ operon mutations have been detected among *E. faecium* and *E. lactis* strains, but their role in CHX susceptibility has not been investigated (Duarte et al., 2019).

The action of 2CSs is to regulate the expression of effector genes in response to environmental cues (Guzman Prieto et al., 2017). The regulon associated with the 2CS-CHX^T^ operon in *E. faecium* Aus0004 has been predicted to include genes involved in peptidoglycan homeostasis, protein, glycerol, or amino sugars metabolism, protection against cationic compounds, and oxidative stress response, among others (Duarte et al., 2019), although confirmatory studies are lacking. Thus, a diverse multi-process cellular response may be prompted by CHX stress (Duarte et al., 2019).

## 7 Co- and cross-resistance between cationic biocides and other antimicrobials

Although there are several differences between CBs and antibiotics (e.g., spectrum and mechanism of action, ratio of in-use concentration per microorganisms’ MICs, commercialized formulations), both have been used for their antimicrobial activity for decades now and CBs have been critical for limiting the need of antibiotic use (Fox et al., 2022). However, research has been suggesting that exposure to biocides may directly or indirectly select for bacterial populations with particular genotypes or phenotypes leading to co- or cross-resistance to antibiotics (Maillard, 2018). *Enterococcus*’ response after exposure to CBs has been shown not only to alter the expression of genes involved in multidrug efflux, bacterial metabolism, and cell wall permeability, which may affect the susceptibility of the cells to very diverse antimicrobials, but also to upregulate genes directly associated with resistance to antibiotics (Bhardwaj et al., 2016; Li and Palmer, 2018). Bhardwaj et al. (2017) found that CHX stress induced the expression of the VanA-type vancomycin resistance genes and genes associated with decreased susceptibility to daptomycin (*liaXYZ*) in *E. faecium*. However, vancomycin susceptibility was actually increased for the VanA-positive *E. faecium* in the presence of subinhibitory concentrations of CHX, revealing a CHX-vancomycin synergy (Bhardwaj et al., 2017, 2021). Excess of D-lactate contributed to this synergism, whereas the deletion of the gene *ddcP*, encoding a membrane-bound carboxypeptidase, and a mutation (S199L) on an ATPase of phosphate-specific transporter, encoded by gene *pstB*, conferred a survival advantage in the presence of both antimicrobials (Bhardwaj et al., 2017, 2021). Nonetheless, the occurrence of a possible cross-resistance between vancomycin and CBs, including CHX, is still controversial. MICs and MBCs of CHX and BC of vancomycin-resistant *Enterococcus* isolates significantly higher than those of vancomycin-susceptible *Enterococcus* spp. have been described among diverse collections (Alotaibi et al., 2017; Ignak et al., 2017; Sobhanipoor et al., 2021). On the contrary, a decreased susceptibility in vancomycin-susceptible *Enterococcus* compared to vancomycin-resistant *Enterococcus* as well as no significant difference between the two groups has also been found for the biocides BC, CHX, DDAC or CPC (Baillie et al., 1992; Barry et al., 1999; Suller and Russell, 1999; Kõljalg et al., 2002; Braga et al., 2013; Roedel et al., 2020; Sobhanipoor et al., 2021). Such disparities among studies might be associated with the different methodologies used, various levels of previous exposure to CBs by the bacteria, or with other genetic or metabolic properties specific to each of the local bacterial populations studied.

A possible link between antibiotic resistance and CBs susceptibility has also been observed for other antibiotics. A decreased susceptibility to CHX and DDAC was detected in *Enterococcus* that were ampicillin resistant or with a high-level of resistance to the aminoglycosides gentamycin and streptomycin (Schwaiger et al., 2014; Wieland et al., 2017; Sobhanipoor et al., 2021). Despite the suggestion of a correlation by these results, no genetic or cellular changes supporting such co-occurring phenotypes have been explored yet.

The selection of antibiotic resistant subpopulations after serial exposure to subinhibitory concentrations of several CBs has been identified (Supplementary Table 2; Bhardwaj et al., 2017; Gadea et al., 2017a,b). Adaptation to CHX, CPC, BC or CE of *E. casseliflavus*, *E. durans*, *E. faecalis, E. faecium, E. saccharolyticus* or other *Enterococcus* spp. led to cross-resistance to the clinically-relevant ampicillin, ciprofloxacin, daptomycin, imipenem and tetracycline (Supplementary Table 2; Bhardwaj et al., 2017; Gadea et al., 2017a,b). On the other hand, there was also evidence of loss of resistance to ampicillin in CHX-adapted *E. faecalis*, to ciprofloxacin in CE-adapted *E. faecium*, and to ceftazidime and/or cefotaxime in BC-adapted *E. faecium*, CPC-adapted *Enterococcus* spp., CE-adapted *E. casseliflavus*, *E. faecium* and *Enterococcus* spp., and CHX-adapted *E. faecium* (Gadea et al., 2017a,b). The genetic mechanisms or phenotypic expression events behind the increase or loss of antibiotic resistance detailed in these studies are mostly unknown, but they were mainly transient and may be associated with non-specific membrane permeability increase, metabolic changes (e.g., decreased growth rate), or others (Gadea et al., 2017a,b). For *E. faecium* 1,231,410, in which decreased susceptibility to daptomycin arose with passages in increasing CHX concentrations, Bhardwaj et al. (2017) detected physiological and genetic alterations in the adapted strains compared to the parental strain. These included significantly lower growth rates, changes in cellular membrane phospholipid and glycolipid content, overexpression of the three-component regulatory system encoded *liaXYZ* involved in cell envelope homeostasis, and mutations in genes associated with global nutritional stress response (*relA*), nucleotide metabolism (*cmk*), multidrug efflux (*efrE*), phosphate acquisition (*phoU*), and glycolipid biosynthesis (*bgsB*) (Bhardwaj et al., 2017). Daptomycin is a lipopeptide antibiotic often used to treat vancomycin-resistant *Enterococcus* spp. infections, that interacts primarily with the bacterial cell membrane, as part of a daptomycin-calcium complex, ultimately leading to cell death (Mishra et al., 2012; Bhardwaj et al., 2017). Thus, the selection of potential structural changes in the biophysical properties of the cell wall or membrane and in the cellular stress responses by the biocide are strongly associated with decreased susceptibility to daptomycin in *Enterococcus* (Mishra et al., 2012; Bhardwaj et al., 2017). Since this antibiotic is used for the treatment of serious *Enterococcus* infections that lack other therapeutic alternatives, and CBs are extensively used in hospitals, these results may have serious clinical implications and deserve further studies (Bhardwaj et al., 2017). However, as previously discussed, it must be taken into consideration that *in vitro* adaptation experiments may not accurately mimic real environments’ conditions in the different contexts.

Of note, CBs, antibiotics, and other antimicrobials such as metals may co-exist as selective agents in many ecosystems, not only in human or animal clinical contexts, the community or food production, but also in wastewater and others (Pal et al., 2015; Wales and Davies, 2015; Singer et al., 2016). In a report produced by SCENIHR (Scientific Committee on Emerging and Newly Identified Health Risks), in 2009, it was stated that ‘biocides are likely to contribute to maintaining selective pressure allowing the presence of mobile genetic elements harboring specific genes involved in the resistance to biocides and antibiotics’, and recommended the surveillance of levels of biocide resistance (Scientific Committee on Emerging and Newly Identified Health Risks [SCENIHR], 2009). Indeed, co-location of genetic determinants conferring decreased susceptibility to CBs and metals and resistance to antibiotics on the same plasmid or other mobile genetic element has been observed among diverse *Enterococcus* spp. strains (Pal et al., 2015; Silveira et al., 2015; Yuan et al., 2018; Pereira et al., 2023). In previous studies, the analyzed genetic contexts of several genes encoding decreased susceptibility to CBs (*qacA/B, qacC, qacZ, oqxAB*) in *Enterococcus* spp., from different sources, geographical regions, or dates of isolation, harbored genes conferring resistance to aminoglycosides, beta-lactams, macrolides, lincosamides, streptogramin of group B, or trimethoprim (Silveira et al., 2015; Yuan et al., 2018; Pereira et al., 2023). Genes coding for decreased susceptibility to metals, namely to copper and cadmium, were also detected within the vicinity of *qacA/B* genes in two clinical *E. faecalis* (Pereira et al., 2023). The genetic contexts of *qacA/B, qacC, qacJ, qacZ, qrg, bcrABC* and *oqxAB* detected in *Enterococcus* were compared with those from other taxa and found to be generally very diverse, probably resulting from a high number of recombination events, as suggested by the abundance of insertion sequences and recombinases detected (Pereira et al., 2023). Also, the co-location of these genes on mobile genetic elements such as plasmids, often carrying toxin-antitoxin systems that contribute to their maintenance in the bacterial populations, may facilitate their spread and, thus, mechanisms of co-selection (Pal et al., 2015; Silveira et al., 2015; Yuan et al., 2018; Fox et al., 2022; Pereira et al., 2023; Maillard and Pascoe, 2024).

Besides co-location of diverse antimicrobial resistance genes on the same genetic contexts, the occurrence of cross-resistance, where a single resistance mechanism to a certain antimicrobial also affects additional compounds, has also been hypothesized in *Enterococcus* spp. (Pal et al., 2015). Genes encoding decreased susceptibility to CBs, such as *emeA, efrEF*, *oqxAB* and *chtR*, are known to be involved in antibiotic resistance, as previously mentioned (Jonas et al., 2001; Lee et al., 2003b; Lubelski et al., 2007; Hürlimann et al., 2016; Yuan et al., 2018). For instance, the *chtR* and *chtS E. faecium* deletion mutants showed an increased susceptibility to both CHX and the antibiotic bacitracin (Guzman Prieto et al., 2017). Additionally, Yuan et al. (2018) proposed that the extensive use of quinoxalines in animal husbandry in China could be selecting for a local high prevalence of the *oqxAB* genes, known to confer decreased susceptibility to such antibiotics and CBs, among *Enterococcus* spp.

Recently, a few studies have proposed another process in which CBs exposure could contribute to antibiotic resistance spread (Zhang et al., 2017; Jutkina et al., 2018; Han et al., 2019; Schmidt et al., 2022; Liu et al., 2023). In these, subinhibitory concentrations of different CBs, including QACs and CHX, have been shown to increase horizontal gene transfer via conjugation, through multiple cellular processes such as increased reactive oxygen species (ROS) production, upregulated stress and SOS response, enhanced cell membrane permeability, and changes in the expression of conjugative transfer genes, among others (Zhang et al., 2017; Jutkina et al., 2018; Han et al., 2019; Schmidt et al., 2022; Liu et al., 2023). However, this has not yet been studied for *Enterococcus* spp.

All these examples identify possible overlaps between responses to different biocides and antibiotics and the potential for the development of co- and cross-resistance among antimicrobials in various environments, with impact in diverse Public Health contexts. However, it is crucial to recognize the large limitations of the research on this topic. Although some studies support the associations detected between decreased susceptibility to CBs and resistance to specific antibiotics through robust methodological approaches, for most, caution is required in interpreting the correlations made as they may be linked to independent, co-occurring events of cellular response to diverse antimicrobials. Thus, the underlying processes by which exposure to one substance may lead to decreased susceptibility to another remain unclear.

## 8 Future perspectives

Currently, the available literature offers valuable insights concerning the state of the genotypic and phenotypic *Enterococcus* spp. susceptibility to CBs, pointing to an effective biocidal activity with still no descriptions of resistance to CB’s typical in-use concentrations. However, it also reveals several key research gaps that need to be tackled in future investigations in *Enterococcus* spp., that could extend to other bacterial species.

Urgent priorities include standardizing biocide susceptibility methodologies that are of relevance to real-world scenarios, facilitating direct study comparisons and the establishment of surveillance protocols applicable across diverse environments. Designing evidence-supported methodologies for this purpose is currently difficult, as the influence of several factors mentioned throughout this review on the activity of biocides against *Enterococcus* spp. or other bacterial species is scarcely studied. Thus, future studies should primarily elucidate the impact of such factors, including different environmental parameters (e.g., temperature, pH, oxygen or nutrient availability), times of biocide exposure, presence of other compounds usually included in biocidal formulations or in the environment, phase of bacterial growth, planktonic cells or biofilms, among others. Furthermore, the integration of cutting-edge technologies into future studies or surveillance programs could facilitate the monitoring of CBs efficacy and susceptibility trends, enabling timely interventions if needed, particularly in settings where CBs are heavily used. Specifically, large longitudinal metadata analyses incorporating WGS and transcriptomic approaches will be critical for clarifying the dynamics of *Enterococcus* spp. populations exposed to CBs, representative of multiple clones and epidemiological backgrounds, and the long-term consequences for biocidal or antibiotic resistance.

Another priority concerns the characterization of the mechanisms involved in *Enterococcus* adaptation to CBs or in the co-selection or cross-resistance with antibiotics. Conducting functional genetic assays, such as the deletion and complementation of genes accompanied by the observation of phenotype changes, as well as the use of high-throughput screening platforms or advanced bioinformatic tools, will be crucial for elucidating the role of genes with a predicted effect on decreased susceptibility to CBs in *Enterococcus* spp. or to better identify still undetected molecular processes. These data will determine the need for strategies aimed at identifying and controlling bacteria harboring particular genotypes in critical contexts.

Fulfilling these methodological and knowledge gaps while taking into account interdisciplinary data from fields such as environmental (e.g., ecotoxicology) and social (e.g., health economics) sciences will provide holistic insights into the intricate dynamics of CBs use and *Enterococcus*’ antimicrobial resistance development. Collaborative efforts among diverse stakeholders at local and global levels across sectors can enable the development of effective One Health strategies that ensure the continued efficacy of these critical agents in safeguarding Public Health.

## Author’s note

All authors are active members of the ESCMID Study Group on Food- and Water-borne Infections (EFWISG).

## Author contributions

AP: Conceptualization, Writing – original draft, Writing – review and editing, Visualization. PA: Writing – review and editing, Funding acquisition. LP: Supervision, Writing – review and editing, Funding acquisition. AF: Writing – review and editing, Supervision, Funding acquisition. CN: Conceptualization, Writing – original draft, Writing – review and editing, Supervision, Funding acquisition.

## Footnotes

1
http://www.bacterio.net/enterococcus.html#r
