# Supplementary material for: Current insights into the effects of cationic biocides exposure on Enterococcus spp
Source: Front Microbiol. 2024 Jun 25;15:1392018. doi: 10.3389/fmicb.2024.1392018 (PMC11242571; doi:10.3389/fmicb.2024.1392018)
Supplement: Supplementary file 1 [file Data_Sheet_1.PDF]

Table S1 - Distribution of cationic biocides MICs and MBCs for *Enterococcus* spp. with different epidemiological and genetic backgrounds.

Biocide class	Biocide	Species	n of isolates	Source	Country	Year	Sequence Type (ST)	Genotypes [1]	Methodology (guidelines [2])	MIC (mg/L)	MBC (mg/L)	Reference
**Quaternary Ammonium Compounds**	**Benzalkonium chloride**	*E. casseliflavus*	7	Food chain (dust samples from breeding pig facilities)	Portugal	2008			BM (CLSI)	2 - 4		Braga et al., 2013
		E. casseliflavus	1	Food chain (milk, cheese)	Portugal			*qacZ*	BM (CLSI)	4		Braga et al., 2011
		E. casseliflavus	2	Food chain (organic foods)	Spain				BM	0.1 - 0.2		Gadea et al., 2017a
		E. durans	2	Food chain (milk, cheese)	Portugal			*qacZ*	BM (CLSI)	4		Braga et al., 2011
		E. durans	3	Food chain (milk, cheese)	Portugal				BM (CLSI)	4		Braga et al., 2011
		E. durans	1	Food chain (organic foods)	Spain				BM	0.5		Gadea et al., 2017a
		E. faecalis ATCC 29212	1	Human infection (urine)	USA		30		BM (CLSI)	8	16	Kouidhi et al., 2011
		E. faecalis ATCC 29212	1	Human infection (urine)	USA		30		BM	8		Yamamoto et al., 2016
		E. faecalis ATCC 29212	1	Human infection (urine)	USA		30		BM (CLSI)	2 - 4		Roedel et al., 2020
		E. faecalis ATCC 29212	1	Human infection (urine)	USA		30		BM (CLSI)	1 - 2	2 - 4	Pereira et al., 2023
		E. faecalis V583	1	Human infection	USA	1987	6	*qacZ*	BM (CLSI)	4	4	Pereira et al., 2023
		E. faecalis	26	Human infection	Portugal, Tunisia	1999-2020	2, 6, 9, 16, 22, 25, 30, 55, 59, 64, 116, 133, 159, 191, 200, 631, 1105, 1165		BM (CLSI)	1 - 4	2 - 4	Pereira et al., 2023
		E. faecalis	55	Human infection	Iran	2018-2020			AD (CLSI)	4 - 16		Kheljan et al., 2022
		E. faecalis	12	Human infection (urinary, vagina, blood culture, pus, catheter, sputum)	Portugal			*qacZ*	BM (CLSI)	2 - 4		Braga et al., 2011
		E. faecalis	6	Human infection (urinary, vagina, blood culture, pus, catheter, sputum)	Portugal				BM (CLSI)	2 - 4		Braga et al., 2011
		E. faecalis B281	1	Human infection (child dental carie)	Tunisia				BM (CLSI)	256	>256	Kouidhi et al., 2011
		E. faecalis 19-47-23	1	Human infection	Germany				BM (CLSI)	4 - 8		Roedel et al., 2020
		E. faecalis	3	Human colonization (faeces of patients at hospital admission)	Brasil	2015-2016	6, 525		BM (CLSI)	2	2 - 4	Pereira et al., 2023
		E. faecalis	4	Human colonization (faeces of long-term care patients)	Portugal	2015-2016	25, 143, 398, 679		BM (CLSI)	2	2 - 4	Pereira et al., 2023
		E. faecalis	21	Human colonization (healthy humans' faeces, urinary tract, breast milk)	Portugal, Angola	2001-2018	21, 30, 40, 63, 64, 81, 116, 179, 191, 200, 206, 209, 275, 308, 394		BM (CLSI)	1 - 2	2 - 4	Pereira et al., 2023
		E. faecalis	45	Human colonization (healthy humans)	Iran	2018-2020			AD (CLSI)	2 - 16		Kheljan et al., 2022
		E. faecalis	49	Pet infection (dogs, cats)	Germany	2017-2019			BM	1.25 - 5		Feßler et al., 2022
		E. faecalis	11	Pet infection (dogs, cats)	Portugal				BM (CLSI)	2 - 4		Braga et al., 2011
		E. faecalis	2	Pet colonization (cat and bird faeces)	Tunisia	2014-2015	21, 116		BM (CLSI)	1	2 - 4	Pereira et al., 2023
		E. faecalis	22	Food chain (poultry carcass, trout, bovine and goat milk, bovine meat, raw meat frozen pet food)	Portugal, Tunisia	1999-2020	21, 27, 32, 34, 40, 49, 117, 141, 200, 202, 206, 209, 227, 288, 436, 674, 721, 843, 860, 1008, 1106		BM (CLSI)	1 - 2	2 - 4	Pereira et al., 2023
		E. faecalis	14	Food chain (aquaculture, piggery, poultry, ovine faeces)	Portugal, Tunisia	2006-2015	21, 22, 35, 40, 59, 65, 139, 200, 209, 330, 386, 631, 749, 872		BM (CLSI)	1 - 2	2 - 4	Pereira et al., 2023

Table S1 (continued)- Distribution of cationic biocides MICs and MBCs for *Enterococcus* spp. with different epidemiological and genetic backgrounds.

Biocide class	Biocide	Species	n of isolates	Source	Country	Year	Sequence Type (ST)	Genotypes [1]	Methodology (guidelines [2])	MIC (mg/L)	MBC (mg/L)	Reference
**Quaternary Ammonium Compounds**	**Benzalkonium chloride**	*E. faecalis*	53	Food chain (dust samples from breeding pig facilities)	Portugal	2008			BM (CLSI)	2 - 4		Braga et al., 2013
		E. faecalis	52	Food chain (pigs and broilers faeces)	Denmark				BM (CLSI)	2 - 8		Aarestrup and Hasman, 2004
		E. faecalis	5	Food chain (swine meat production chain)	Italy			*emeA*	BM (CLSI)	2 - 4		Rizzotti et al., 2016
		E. faecalis	4	Food chain (swine meat production chain)	Italy				BM (CLSI)	4		Rizzotti et al., 2016
		E. faecalis	8	Food chain (cheese)	Portugal	2022			BM (CLSI)	5 - 20	10 - 20	Salamandane et al., 2023
		E. faecalis	12	Food chain (milk, cheese)	Portugal			*qacZ*	BM (CLSI)	4		Braga et al., 2011
		E. faecalis	5	Food chain (milk, cheese)	Portugal				BM (CLSI)	4		Braga et al., 2011
		E. faecalis	38	Food chain (traditional fermented foods)	Morocco, Spain, and Republic of Congo				BM (CLSI)	<0.1		Lavilla Lerma et al., 2014
		E. faecalis	2	Food chain (organic foods)	Spain				BM	0.05 - 0.5		Gadea et al., 2017a
		E. faecalis	4	Food chain (ready-to-eat salads)	Portugal	2010	141, 165, 309, 594		BM (CLSI)	1 - 2	4	Pereira et al., 2023
		E. faecalis	3	Environment (hospital sewage)	Portugal	2002	16, 35, 49		BM (CLSI)	2	2 - 4	Pereira et al., 2023
		E. faecalis	34	Environment (hospital sewage)	Iran	2018-2020			AD (CLSI)	4 - 16		Kheljan et al., 2022
		E. faecalis	3	Environment (urban wastewater treatment plant)	Tunisia	2014-2015	23, 86, 117		BM (CLSI)	1 - 2	2 - 4	Pereira et al., 2023
		E. faecalis	21	Environment (municipal sewage)	Iran	2018-2020			AD (CLSI)	8 - 16		Kheljan et al., 2022
		E. faecalis	38	Environment (poultry sewage)	Iran	2018-2020			AD (CLSI)	2 - 16		Kheljan et al., 2022
		E. faecalis	29	Environment (livestock sewage)	Iran	2018-2020			AD (CLSI)	4 - 16		Kheljan et al., 2022
		E. faecalis	2	Environment (river)	Portugal	2003	1, 4		BM (CLSI)	2	2 - 4	Pereira et al., 2023
		E. faecalis	56		Spain	2001-2009			BM (CLSI)	1 - 8	2 - 16	Morrissey et al., 2014
		E. faecalis	1						BM	2	3.3	Cowley et al., 2015
		E. faecium Aus0004	1	Human infection (blood)	Australia	1998			BM (EUCAST)	4	8	Dejoies et al., 2021
		E. faecium	26	Human infection	Portugal, Spain, Tunisia	1996-2020	17, 18, 22, 78, 80, 117, 190, 280, 391, 412, 419, 494, 515, 780		BM (CLSI)	1 - 4	2 - 4	Pereira et al., 2023
		E. faecium	12	Human infection (blood)	Denmark	2004	22, 95, 178, 296, 361, 372, 697, 1277, 1279		BM (CLSI)	2 - 8	2 - 8	Alotaibi et al., 2017
		E. faecium	37	Human infection (blood, urine, tissue)	Denmark	2012-2013	18, 80, 117, 203		BM (CLSI)	4 - 8	4 - 16	Alotaibi et al., 2017
		E. faecium	17	Human infection	Iran	2018-2020			AD (CLSI)	8 - 16		Kheljan et al., 2022
		E. faecium	5	Human infection (urinary, vagina, blood culture, pus, catheter, sputum)	Portugal			*qacZ*	BM (CLSI)	4 - 8		Braga et al., 2011
		E. faecium	5	Human infection (urinary, vagina, blood culture, pus, catheter, sputum)	Portugal				BM (CLSI)	4 - 8		Braga et al., 2011

Table S1 (continued)- Distribution of cationic biocides MICs and MBCs for *Enterococcus* spp. with different epidemiological and genetic backgrounds.

Biocide class	Biocide	Species	n of isolates	Source	Country	Year	Sequence Type (ST)	Genotypes [1]	Methodology (guidelines [2])	MIC (mg/L)	MBC (mg/L)	Reference
**Quaternary Ammonium Compounds**	**Benzalkonium chloride**	*E. faecium*	3	Human infection (blood)	Germany				BM (CLSI)	8 - 16		Roedel et al., 2020
		E. faecium	90	Human infection (blood cultures, swabs, urine, faeces)	Germany				BM (Vacuum Dried Biocide Microtiter Plates)	1 - 8	8 - 16	Roedel et al., 2020
		E. faecium	4	Human colonization (faeces of long-term care patients)	Portugal	2015-2016	80, 117, 262		BM (CLSI)	2 - 4	2 - 4	Pereira et al., 2023
		E. faecium	15	Human colonization (healthy humans' faeces, vagina)	Portugal, Angola	2001-2017	6, 8, 18, 22, 32, 89, 147, 845, 1051, 2095		BM (CLSI)	1 - 4	1 - 4	Pereira et al., 2023
		E. faecium	219	Human colonization (healthy humans)	Iran	2018-2020			AD (CLSI)	4 - 32		Kheljan et al., 2022
		E. faecium	37	Pet infections (dogs, cats)	Germany	2017-2019			BM	1.25 - 5		Feßler et al., 2022
		E. faecium	4	Pet infections (dogs, cats)	Portugal				BM (CLSI)	4 - 8		Braga et al., 2011
		E. faecium	27	Food chain (poultry carcass, trout, bovine milk, bovine meat, raw meat frozen pet food)	Portugal, Tunisia	1999-2020	9, 12, 17, 18, 25, 27, 32, 80, 157, 203, 245, 264, 451, 683, 1058, 1059, 1091, 1263, 2092		BM (CLSI)	1 - 4	1 - 4	Pereira et al., 2023
		E. faecium	18	Food chain (aquaculture, piggery, poultry faeces)	Portugal, Angola	2001-2020	5, 6, 29, 30, 94, 101, 132, 185, 264, 393, 430, 434, 891, 970, 971		BM (CLSI)	0.5 - 2	1 - 4	Pereira et al., 2023
		E. faecium	22	Food chain (dust samples from breeding pig facilities)	Portugal	2008			BM (CLSI)	2 - 4		Braga et al., 2013
		E. faecium	78	Food chain (pigs, broilers and cattle faeces)	Denmark				BM (CLSI)	2 - 16		Aarestrup and Hasman, 2004
		E. faecium CF3 1.3	1	Food chain (ceca of an adult broiler chicken)					BM (CLSI)	4		Beier et al., 2008
		E. faecium	79	Food chain (farm animal's faeces)					BM	25		Valenzuela et al., 2013a
		E. faecium	1	Food chain (swine meat production chain)	Italy			*qacA/B*, *emeA*	BM (CLSI)	4		Rizzotti et al., 2016
		E. faecium	8	Food chain (swine meat production chain)	Italy			*emeA*	BM (CLSI)	2 - 4		Rizzotti et al., 2016
		E. faecium	3	Food chain (swine meat production chain)	Italy				BM (CLSI)	2 - 4		Rizzotti et al., 2016
		E. faecium	8	Food chain (cheese)	Portugal	2022			BM (CLSI)	5 - 20	10 - 40	Salamandane et al., 2023
		E. faecium	3	Food chain (milk, cheese)	Portugal			*qacZ*	BM (CLSI)	4		Braga et al., 2011
		E. faecium	84	Food chain (traditional fermented foods)	Morocco, Spain, and Republic of Congo				BM (CLSI)	<0.1		Lavilla Lerma et al., 2014
		E. faecium	13	Food chain (organic foods)	Spain				BM	0.1 - 0.5		Gadea et al., 2017a
		E. faecium	4	Food chain (ready-to-eat salads)	Portugal	2010	352, 640, 666		BM (CLSI)	1 - 2	1 - 4	Pereira et al., 2023
		E. faecium	20	Food chain (fresh produce)	Spain		22, 26, 43, 46, 55, 94, 296		BM	12.5 - 50		Burgos et al., 2014
		E. faecium	2	Environment (hospital sewage)	Portugal	2001-2002	368, 2091		BM (CLSI)	2	2	Pereira et al., 2023
		E. faecium E241	1	Environment (hospital sewage)	Portugal	2002	17	*qacZ*	BM (CLSI)	4	4	Pereira et al., 2023
		E. faecium E241	1	Environment (hospital sewage)	Portugal	2002	17	*qacZ*	AD (CLSI)	9		Silveira et al., 2015
		E. faecium	63	Environment (hospital sewage)	Iran	2018-2020			AD (CLSI)	2 - 32		Kheljan et al., 2022

Table S1 (continued)- Distribution of cationic biocides MICs and MBCs for *Enterococcus* spp. with different epidemiological and genetic backgrounds.

Biocide class	Biocide	Species	n of isolates	Source	Country	Year	Sequence Type (ST)	Genotypes [1]	Methodology (guidelines [2])	MIC (mg/L)	MBC (mg/L)	Reference
**Quaternary Ammonium Compounds**	**Benzalkonium chloride**	*E. faecium*	3	Environment (urban wastewater treatment plant)	Tunisia	2014-2015	666		BM (CLSI)	1 - 2	2	Pereira et al., 2023
		E. faecium	42	Environment (municipal sewage)	Iran	2018-2020			AD (CLSI)	4 - 16		Kheljan et al., 2022
		E. faecium	50	Environment (community waste water)	USA				BM (CLSI)	2 - 8		Beier et al., 2008
		E. faecium	39	Environment (poultry sewage)	Iran	2018-2020			AD (CLSI)	2 - 16		Kheljan et al., 2022
		E. faecium	45	Environment (livestock sewage)	Iran	2018-2020			AD (CLSI)	4 - 16		Kheljan et al., 2022
		E. faecium	3	Environment (river)	Portugal	2003	22, 132, 2093		BM (CLSI)	2	2 - 4	Pereira et al., 2023
		E. faecium	2	Others (wild birds)	Germany, Canada	2011-2013	78, 448		BM (CLSI)	1 - 4	2 - 4	Pereira et al., 2023
		E. faecium	53			1986-2009			BM (CLSI)	1 - 8	2 - 16	Morrissey et al., 2014
		E. gallinarum	2	Food chain (dust sample from breeding pig facilities)	Portugal	2008			BM (CLSI)	2 - 4		Braga et al., 2013
		E. hirae ATCC 10541	1						BM	8		Yamamoto et al., 2016
		E. hirae	2	Human infection (urinary, vagina, blood culture, pus, catheter, sputum)	Portugal			*qacZ*	BM (CLSI)	2 - 4		Braga et al., 2011
		E. hirae	39	Food chain (dust samples from breeding pig facilities)	Portugal	2008			BM (CLSI)	1 - 4		Braga et al., 2013
		E. hirae	1	Food chain (milk, cheese)	Portugal			*qacZ*	BM (CLSI)	4		Braga et al., 2011
		E. raffinosus	2	Food chain (dust samples from breeding pig facilities)	Portugal	2008			BM (CLSI)	1 - 2		Braga et al., 2013
		E. solitarius	1	Pet infection (dogs, cats)	Portugal				BM (CLSI)	2		Braga et al., 2011
		Enterococcus spp.	69	Human infection (blood, cerebrospinal fluid, urine, abscess, catheter tips, nasal secretions, endotracheal aspiration fluid)		2010-2011			AD (CLSI)	8 - 16		Ignak et al., 2017
		Enterococcus spp.	59	Human infection					BM	25 - 250		Valenzuela et al., 2013a
		Enterococcus spp.	54	Human infection					BM	2 - 8		Sobhanipoor et al., 2021
		Enterococcus spp.	50	Human colonization (healthy humans' faeces)					BM	2 - 16		Sobhanipoor et al., 2021
		Enterococcus spp.	500	Food chain (broilers, cattle and pigs)	Denmark	1995-1996			BM	<30		Sidhu et al., 2002
		Enterococcus spp.	25	Food chain (dust samples from breeding pig facilities)	Portugal	2008			BM (CLSI)	<0.25 - 4		Braga et al., 2013
		Enterococcus spp.	25	Food chain (seafood)					BM	25		Valenzuela et al., 2013a
		Enterococcus spp.	40	Food chain (meat and dairy products)					BM	2.5 - 250		Valenzuela et al., 2013a
		Enterococcus spp.	6	Food chain (organic foods)	Spain				BM	0.1 - 0.5		Gadea et al., 2017a
		Enterococcus spp.	22	Food chain (vegetables)					BM	25		Valenzuela et al., 2013a
		Enterococcus spp.	5	Environment (samples of homes of antibacterial product users)	USA, UK				BM (CLSI)	0.426 - 0.851		Cole et al., 2003
		Enterococcus spp.	6	Environment (samples of homes of antibacterial product nonusers)	USA, UK				BM (CLSI)	0.426 - 0.851		Cole et al., 2003
		Enterococcus spp.	47	Others (wildflowers)					BM	25 - 250		Valenzuela et al., 2013a
		Enterococcus spp.	5						AD	5 - 6		Suller and Russel, 1999

Table S1 (continued)- Distribution of cationic biocides MICs and MBCs for *Enterococcus* spp. with different epidemiological and genetic backgrounds.

Biocide class	Biocide	Species	n of isolates	Source	Country	Year	Sequence Type (ST)	Genotypes [1]	Methodology (guidelines [2])	MIC (mg/L)	MBC (mg/L)	Reference
**Quaternary Ammonium Compounds**	**Cetylpyridinium chloride**	*E. casseliflavus*	2	Food chain (organic foods)	Spain				BM	0.5 - 1		Gadea et al., 2017a
		E. durans	1	Food chain (organic foods)	Spain				BM	5		Gadea et al., 2017a
		E. faecalis ATCC 29212	1	Human infection (urine)	USA		30		BM (CLSI)	2 - 4	-	Roedel et al., 2020
		E. faecalis ATCC 29212	1	Human infection (urine)	USA		30		BM	1	4	Lv et al., 2023
		E. faecalis 19-47-23	1	Human infection	Germany				BM (CLSI)	2 - 4	-	Roedel et al., 2020
		E. faecalis	38	Food chain (traditional fermented foods)	Morocco, Spain, and Republic of Congo				BM (CLSI)	<0.1		Lavilla Lerma et al., 2014
		E. faecalis	2	Food chain (organic foods)	Spain				BM	1 - 5		Gadea et al., 2017a
		E. faecalis SS497	1						BM	2		Kitagawa et al., 2016
		E. faecium	3	Human infection (blood)	Germany				BM (CLSI)	2 - 8	-	Roedel et al., 2020
		E. faecium	90	Human infection (blood cultures, swabs, urine, faeces)	Germany				BM (Vacuum Dried Biocide Microtiter Plates)	≤1 - 4	4	Roedel et al., 2020
		E. faecium	79	Food chain (farm animal's faeces)					BM	2.5 - 25		Valenzuela et al., 2013a
		E. faecium	84	Food chain (traditional fermented foods)	Morocco, Spain, and Republic of				BM (CLSI)	<0.1		Lavilla Lerma et al., 2014
		E. faecium	20	Food chain (fresh produce)	Spain		22, 26, 43, 46, 55, 94, 296		BM	1 - 2	-	Burgos et al., 2014
		E. faecium	13	Food chain (organic foods)	Spain				BM	0.5 - 5		Gadea et al., 2017a
		Enterococcus spp.	59	Human infection					BM	2.5 - 250		Valenzuela et al., 2013a
		Enterococcus spp.	40	Food chain (meat and dairy foods)					BM	2.5 - 25		Valenzuela et al., 2013a
		Enterococcus spp.	25	Food chain (seafood)					BM	2.5 - 25		Valenzuela et al., 2013a
		Enterococcus spp.	22	Food chain (vegetables)					BM	2.5 - 25		Valenzuela et al., 2013a
		Enterococcus spp.	6	Food chain (organic foods)	Spain				BM	1 - 50		Gadea et al., 2017a
		Enterococcus spp.	47	Others (wildflowers)					BM	2.5 - 25		Valenzuela et al., 2013a
		Enterococcus spp.	5						AD	5 - 6		Suller and Russel, 1999
	**Cetrimide**	*E. casseliflavus*	2	Food chain (organic foods)	Spain				BM	5 - 10		Gadea et al., 2017b
		E. durans	1	Food chain (organic foods)	Spain				BM	1		Gadea et al., 2017b
		E. faecalis WIBG 1.1	1	Human infection (wound)					BM	12.1	29	Forbes et al., 2014
		E. faecalis	38	Food chain (traditional fermented foods)	Morocco, Spain, and Republic of Congo				BM (CLSI)	<0.1		Lavilla Lerma et al., 2014
		E. faecalis	2	Food chain (organic foods)	Spain				BM	0.5 - 5		Gadea et al., 2017b
		E. faecium	79	Food chain (farm animal's faeces)					BM	2.5 - 25		Valenzuela et al., 2013a
		E. faecium	84	Food chain (traditional fermented foods)	Morocco, Spain, and Republic of Congo				BM (CLSI)	<0.1		Lavilla Lerma et al., 2014
		E. faecium	20	Food chain (fresh produce)	Spain		22, 26, 43, 46, 55, 94, 296		BM	25 - 75	-	Burgos et al., 2014
		E. faecium	13	Food chain (organic foods)	Spain				BM	0.5 - 10		Gadea et al., 2017b
		Enterococcus spp.	59	Human infection					BM	2.5 - 250		Valenzuela et al., 2013a
		Enterococcus spp.	25	Food chain (seafood)					BM	2.5 - 25		Valenzuela et al., 2013a
		Enterococcus spp.	40	Food chain (meat and dairy foods)					BM	2.5 - 25		Valenzuela et al., 2013a
		Enterococcus spp.	22	Food chain (vegetables)					BM	2.5 - 25		Valenzuela et al., 2013a
		Enterococcus spp.	6	Food chain (organic foods)	Spain				BM	0.1 - 10		Gadea et al., 2017b
		Enterococcus spp.	47	Others (wildflowers)					BM	2.5 - 25		Valenzuela et al., 2013a

Table S1 (continued)- Distribution of cationic biocides MICs and MBCs for *Enterococcus* spp. with different epidemiological and genetic backgrounds.

Biocide class	Biocide	Species	n of isolates	Source	Country	Year	Sequence Type (ST)	Genotypes [1]	Methodology (guidelines [2])	MIC (mg/L)	MBC (mg/L)	Reference
**Quaternary Ammonium Compounds**	**Didecyldimethylammonium chloride**	*E. faecalis* ATCC 29212	1	Human infection (urine)	USA		30		BM (CLSI)	2		Roedel et al., 2020
		E. faecalis ATCC 29212	1	Human infection (urine)	USA		30		BM (CLSI)	0.5 - 1	1 - 2	Pereira et al., 2023
		E. faecalis V583	1	Human infection	USA	1987	6	*qacZ*	BM (CLSI)	2	2	Pereira et al., 2023
		E. faecalis	26	Human infection	Portugal, Tunisia	1999-2020	2, 6, 9, 16, 22, 25, 30, 55, 59, 64, 116, 133, 159, 191, 200, 631, 1105, 1165		BM (CLSI)	1 - 2	1 - 4	Pereira et al., 2023
		E. faecalis	42	Human infection (blood)	Germany	2008-2009			BM	1.05		Bischoff et al., 2012
		E. faecalis Efa-AB-2	1	Human infection (blood)	Germany	2008-2009		*qacA/B*	BM	2.45 – 3.5		Bischoff et al., 2012
		E. faecalis	88	Human infection (blood)	Germany				BM	1.05 - 3.5		Schwaiger et al., 2014
		E. faecalis 19-47-23	1	Human infection	Germany				BM (CLSI)	2 - 4	-	Roedel et al., 2020
		E. faecalis	109	Human colonization (hospitalized patient's faeces)	Germany	2008-2009			BM	1.05		Bischoff et al., 2012
		E. faecalis Efa-smr-1	1	Human colonization (hospitalized patient's faeces)	Germany	2008-2009		*qacC*	BM	1.05		Bischoff et al., 2012
		E. faecalis	210	Human colonization (hospitalized patient's faeces)	Germany				BM	1.05 - 3.5		Schwaiger et al., 2014
		E. faecalis	3	Human colonization (faeces of patients at hospital admission)	Brasil	2015-2016	6, 525		BM (CLSI)	1	2	Pereira et al., 2023
		E. faecalis	102	Human colonization (outpatients' faeces)	Germany				BM	1.05 - 3.5		Schwaiger et al., 2014
		E. faecalis	4	Human colonization (faeces of long-term care patients)	Portugal	2015-2016	25, 143, 398, 679		BM (CLSI)	1	2 - 4	Pereira et al., 2023
		E. faecalis	21	Human colonization (healthy humans' faeces, urinary tract, breast milk)	Portugal, Angola	2001-2018	21, 30, 40, 63, 64, 81, 116, 179, 191, 200, 206, 209, 275, 308, 394		BM (CLSI)	1	1 - 4	Pereira et al., 2023
		E. faecalis	2	Pet colonization (faeces of cats and birds)	Tunisia	2014-2015	21, 116		BM (CLSI)	1	1 - 2	Pereira et al., 2023
		E. faecalis	22	Food chain (poultry carcass, trout, bovine and goat milk, bovine meat, raw meat frozen pet food)	Portugal, Tunisia	1999-2020	21, 27, 32, 34, 40, 49, 117, 141, 200, 202, 206, 209, 227, 288, 436, 674, 721, 843, 860, 1008, 1106		BM (CLSI)	1 - 2	2 - 4	Pereira et al., 2023
		E. faecalis	14	Food chain (aquaculture, piggery, poultry, ovine faeces)	Portugal, Tunisia	2006-2015	21, 22, 35, 40, 59, 65, 139, 200, 209, 330, 386, 631, 749, 872		BM (CLSI)	1 - 2	2 - 4	Pereira et al., 2023
		E. faecalis Efa-AB-1	1	Food chain (cattle)	Germany			*qacA/B*	BM	1.05		Bischoff et al., 2012
		E. faecalis	112	Food chain (meat products)	Germany				BM	1.05		Bischoff et al., 2012
		E. faecalis	226	Food chain (faeces of farm animals)	Germany				BM	1.05		Bischoff et al., 2012
		E. faecalis	54	Food chain (poultry)	Germany				BM	1.05 - 2.1		Schwaiger et al., 2014
		E. faecalis	30	Food chain (beef)	Germany				BM	1.05 - 2.1		Schwaiger et al., 2014
		E. faecalis	28	Food chain (pork)	Germany				BM	1.05 - 2.1		Schwaiger et al., 2014
		E. faecalis	50	Food chain (cattle, matistis milk)	Germany				BM	1.05		Schwaiger et al., 2014
		E. faecalis	76	Food chain (swine faeces)	Germany				BM	1.05		Schwaiger et al., 2014

Table S1 (continued)- Distribution of cationic biocides MICs and MBCs for *Enterococcus* spp. with different epidemiological and genetic backgrounds.

Biocide class	Biocide	Species	n of isolates	Source	Country	Year	Sequence Type (ST)	Genotypes [1]	Methodology (guidelines [2])	MIC (mg/L)	MBC (mg/L)	Reference
**Quaternary Ammonium Compounds**	**Didecyldimethylammonium chloride**	*E. faecalis*	50	Food chain (cattle faeces)	Germany				BM	1.05		Schwaiger et al., 2014
		E. faecalis	50	Food chain (chicken faeces)	Germany				BM	1.05 - 1.75		Schwaiger et al., 2014
		E. faecalis	56	Food chain (poultry caecum, cloacal swab, faeces)	Germany				MD (German Veterinary Society guidelines)	0.14 - 1.44		Wieland et al., 2017
		E. faecalis	12	Food chain (cloacal swabs of hunted chicken)	Finland				MD (German Veterinary Society guidelines)	0.36 - 1.44		Wieland et al., 2017
		E. faecalis	96	Food chain (dairy products)	Germany				BM	1.05		Bischoff et al., 2012
		E. faecalis Efa-smr-2	1	Food chain (cheese ''Camembert'')	Germany			*qacC*	BM	1.05		Bischoff et al., 2012
		E. faecalis	96	Food chain (dairy products)	Germany				BM	1.05 - 3.5		Schwaiger et al., 2014
		E. faecalis	4	Food chain (ready-to-eat salads)	Portugal	2010	141, 165, 309, 594		BM (CLSI)	1	2 - 4	Pereira et al., 2023
		E. faecalis	3	Environment (hospital sewage)	Portugal	2002	16, 35, 49		BM (CLSI)	1	2 - 4	Pereira et al., 2023
		E. faecalis	3	Environment (urban wastewater treatment plant)	Tunisia	2014-2015	23, 86, 117		BM (CLSI)	1	2 - 4	Pereira et al., 2023
		E. faecalis	2	Environment (river)	Portugal	2003	1, 4		BM (CLSI)	1	2 - 4	Pereira et al., 2023
		E. faecalis	1						BM	1	1	Cowley et al., 2015
		E. faecium	26	Human infection	Portugal, Spain, Tunisia	1996-2020	17, 18, 22, 78, 80, 117, 190, 280, 391, 412, 419, 494, 515, 780		BM (CLSI)	1 - 2	1 - 4	Pereira et al., 2023
		E. faecium	45	Human infection (blood)	Germany				BM	1.05 - 1.75		Schwaiger et al., 2014
		E. faecium	90	Human infection (blood cultures, swabs, urine, faeces)	Germany				BM (Vacuum Dried Biocide Microtiter Plates)	≤0.5 - 2	1 - 2	Roedel et al., 2020
		E. faecium	3	Human infection (blood)	Germany				BM (CLSI)	2 - 4	-	Roedel et al., 2020
		E. faecium	52	Human colonization (hospitalized patient's faeces)	Germany				BM	1.05 - 1.75		Schwaiger et al., 2014
		E. faecium	38	Human colonization (outpatients' faeces)	Germany				BM	1.05 - 2.8		Schwaiger et al., 2014
		E. faecium	4	Human colonization (faeces of long-term care patients)	Portugal	2015-2016	80, 117, 262		BM (CLSI)	1 - 2	2 - 4	Pereira et al., 2023
		E. faecium	15	Human colonization (healthy humans' faeces, vagina)	Portugal, Angola	2001-2017	6, 8, 18, 22, 32, 89, 147, 845, 1051, 2095		BM (CLSI)	1 - 2	1 - 2	Pereira et al., 2023
		E. faecium	27	Food chain (poultry carcass, trout, bovine milk, bovine meat, raw meat frozen pet food)	Portugal, Tunisia	1999-2020	9, 12, 17, 18, 25, 27, 32, 80, 157, 203, 245, 264, 451, 683, 1058, 1059, 1091, 1263, 2092		BM (CLSI)	1 - 2	1 - 4	Pereira et al., 2023
		E. faecium	18	Food chain (aquaculture, piggery, poultry faeces)	Portugal, Angola	2001-2020	5, 6, 29, 30, 94, 101, 132, 185, 264, 393, 430, 434, 891, 970, 971		BM (CLSI)	0.5 - 1	1 - 2	Pereira et al., 2023
		E. faecium CF3 1.3	1	Food chain (ceca of an adult broiler chicken)					BM (CLSI)	0.5		Beier et al., 2008
		E. faecium	64	Food chain (poultry caecum and faeces)	Germany				MD (German Veterinary Society guidelines)	0.14 - 1.44		Wieland et al., 2017
		E. faecium	17	Food chain (cloacal swabs of hunted chicken)	Finland				MD (German Veterinary Society guidelines)	0.36 - 1.44		Wieland et al., 2017

Table S1 (continued)- Distribution of cationic biocides MICs and MBCs for *Enterococcus* spp. with different epidemiological and genetic backgrounds.

Biocide class	Biocide	Species	n of isolates	Source	Country	Year	Sequence Type (ST)	Genotypes [1]	Methodology (guidelines [2])	MIC (mg/L)	MBC (mg/L)	Reference
**Quaternary Ammonium Compounds**	**Didecyldimethylammonium chloride**	*E. faecium*	4	Food chain (ready-to-eat salads)	Portugal	2010	352, 640, 666		BM (CLSI)	0.5 - 1	1 - 2	Pereira et al., 2023
		E. faecium	2	Environment (hospital sewage)	Portugal	2001-2002	368, 2091		BM (CLSI)	1 - 2	2	Pereira et al., 2023
		E. faecium E241	1	Environment (hospital sewage)	Portugal	2002	17	*qacZ*	BM (CLSI)	2	2	Pereira et al., 2023
		E. faecium	3	Environment (urban wastewater treatment plant)	Tunisia	2014-2015	666		BM (CLSI)	1	2	Pereira et al., 2023
		E. faecium	50	Environment (community waste water)	USA				BM (CLSI)	0.5 - 2		Beier et al., 2008
		E. faecium	3	Environment (river)	Portugal	2003	22, 132, 2093		BM (CLSI)	1	2	Pereira et al., 2023
		E. faecium	2	Others (wild birds)	Germany, Canada	2011-2013	78, 448		BM (CLSI)	1 - 2	2	Pereira et al., 2023
**Biguanides**	**Chlorhexidine**	*E. avium*	1	Human infection					BM (CLSI)	1		Grare et al., 2010
		E. casseliflavus	1	Human infection					BM (CLSI)	2		Grare et al., 2010
		E. casseliflavus	7	Food chain (dust samples from breeding pig facilities)	Portugal	2008			BM (CLSI)	1 - 4		Braga et al., 2013
		E. casseliflavus	1	Food chain (milk, cheese)	Portugal			*qacZ*	BM (CLSI)	4		Braga et al., 2011
		E. casseliflavus	2	Food chain (organic foods)	Spain				BM	5		Gadea et al., 2017b
		E. durans	2	Food chain (milk, cheese)	Portugal			*qacZ*	BM (CLSI)	4		Braga et al., 2011
		E. durans	3	Food chain (milk, cheese)	Portugal				BM (CLSI)	4 - 8		Braga et al., 2011
		E. durans	1	Food chain (organic foods)	Spain				BM	5		Gadea et al., 2017b
		E. faecalis ATCC 29211	1	Human infection (urine)	USA		30		BM (CLSI)	<1		Grare et al., 2010
		E. faecalis ATCC 29212	1	Human infection (urine)	USA		30		BM (DIN:58940-7, 58940-8)	16	16	Koburger et al., 2010
		E. faecalis ATCC 29212	1	Human infection (urine)	USA		30		MD (CLSI)	32	256	Arslan et al., 2011
		E. faecalis ATCC 29212	1	Human infection (urine)	USA		30		BM	2.5	10	Anand et al., 2015
		E. faecalis ATCC 29212	1	Human infection (urine)	USA		30		BM	27		Yamamoto et al., 2016
		E. faecalis ATCC 29212	1	Human infection (urine)	USA		30		BM (CLSI)	8		Roedel et al., 2020
		E. faecalis ATCC 4083	1						BM	3.9		Wang et al., 2017
		E. faecalis NCTC 775	1						BM (EUCAST)	2	4	Touzel et al., 2016
		E. faecalis	47	Human infection	Portugal, Tunisia	1996-2019	2, 6, 9, 16, 21, 22, 25, 30, 40, 41, 55, 59, 64, 97, 116, 126, 133, 159, 191, 200, 275, 286, 319, 397, 631, 679, 1105, 1164, 1165		BM (CLSI)	1 - 8	4 - 64	Pereira et al., 2022
		E. faecalis	55	Human infection	Iran	2018-2020			AD (CLSI)	2 - 8		Kheljan et al., 2022
		E. faecalis	10	Human infection					MD (CLSI)	4 [3]	24 [3]	Kõljalg et al., 2002
		E. faecalis	2	Human infection					BM (CLSI)	<1 - 2		Grare et al., 2010
		E. faecalis	1	Human infection					BM (DIN:58940-7, 58940-8)	16	32	Koburger et al., 2010

Table S1 (continued)- Distribution of cationic biocides MICs and MBCs for *Enterococcus* spp. with different epidemiological and genetic backgrounds.

Biocide class	Biocide	Species	n of isolates	Source	Country	Year	Sequence Type (ST)	Genotypes [1]	Methodology (guidelines [2])	MIC (mg/L)	MBC (mg/L)	Reference
**Biguanides**	**Chlorhexidine**	*E. faecalis*	12	Human infection (urinary, vagina, blood culture, pus, catheter, sputum)	Portugal			*qacZ*	BM (CLSI)	2 - 4		Braga et al., 2011
		E. faecalis	6	Human infection (urinary, vagina, blood culture, pus, catheter, sputum)	Portugal				BM (CLSI)	2 - 4		Braga et al., 2011
		E. faecalis WIBG 1.1	1	Human infection (wound)					BM	3.6	26.6	Forbes et al., 2014
		E. faecalis 19-47-23	1	Human infection	Germany				BM (CLSI)	8 - 16		Roedel et al., 2020
		E. faecalis	3	Human colonization (faeces of patients at hospital admission)	Brasil	2015-2016	6, 525		BM (CLSI)	4 - 8	8 - 16	Pereira et al., 2022
		E. faecalis	7	Human colonization (faeces of long-term care patients)	Portugal	2015-2016	25, 26, 40, 143, 191, 398, 679		BM (CLSI)	1 - 8	8 - 64	Pereira et al., 2022
		E. faecalis	49	Pet infection (dogs, cats)	Germany	2017-2019			BM	0.3 - 10		Feßler et al., 2022
		E. faecalis	11	Pet infection (dogs, cats)	Portugal				BM (CLSI)	4		Braga et al., 2011
		E. faecalis	3	Pet colonization (dog, cat, bird)	Tunisia	2014-2015	21, 116		BM (CLSI)	4	8 - 32	Pereira et al., 2022
		E. faecalis	44	Human colonization (healthy humans' faeces, urinary tract, vagina, breast milk)	Portugal, Angola	2001-2018	16, 21, 23, 30, 40, 55, 56, 63, 64, 81, 96, 116, 117, 168, 179, 191, 200, 206, 209, 275, 308, 394		BM (CLSI)	0.5 - 8	1 - 64	Pereira et al., 2022
		E. faecalis	45	Human colonization (healthy humans)	Iran	2018-2020			AD (CLSI)	0.5 - 8		Kheljan et al., 2022
		E. faecalis	36	Food chain (poultry carcass, trout, bovine and goat milk, bovine meat, raw meat frozen pet food)	Portugal, Tunisia	1999-2020	4, 16, 21, 22, 25, 27, 30, 32, 34, 40, 49, 68, 82, 117, 141, 200, 202, 206, 209, 227, 249, 288, 436, 648, 674, 721, 843, 860, 1106, 1107, 1008, 1009		BM (CLSI)	0.5 - 8	8 - 64	Pereira et al., 2022
		E. faecalis	23	Food chain (aquaculture, piggery, poultry, ovine faeces)	Portugal, Tunisia	2006-2015	21, 22, 35, 40, 59, 65, 100, 139, 200, 209, 330, 386, 445, 631, 749, 872		BM (CLSI)	1 - 8	8 - 64	Pereira et al., 2022
		E. faecalis	53	Food chain (dust samples from breeding pig facilities)	Portugal	2008			BM (CLSI)	2 - 8		Braga et al., 2013
		E. faecalis	52	Food chain (pigs and broilers faeces)	Denmark				BM (CLSI)	0.5 - 8		Aarestrup and Hasman, 2004
		E. faecalis	5	Food chain (swine meat production chain)	Italy			*emeA*	BM (CLSI)	8 - 12		Rizzotti et al., 2016
		E. faecalis	4	Food chain (swine meat production chain)	Italy				BM (CLSI)	8 - 10		Rizzotti et al., 2016
		E. faecalis	12	Food chain (milk, cheese)	Portugal			*qacZ*	BM (CLSI)	4 - 8		Braga et al., 2011
		E. faecalis	5	Food chain (milk, cheese)	Portugal				BM (CLSI)	4		Braga et al., 2011
		E. faecalis	38	Food chain (traditional fermented foods)	Morocco, Spain, and Republic of Congo				BM (CLSI)	<0.1-2.5		Lavilla Lerma et al., 2014
		E. faecalis	6	Food chain (ready-to-eat salads)	Portugal	2010	100, 141, 165, 309, 579, 594		BM (CLSI)	4	8 - 32	Pereira et al., 2022
		E. faecalis	2	Food chain (organic foods)	Spain				BM	5		Gadea et al., 2017b

Table S1 (continued)- Distribution of cationic biocides MICs and MBCs for *Enterococcus* spp. with different epidemiological and genetic backgrounds.

Biocide class	Biocide	Species	n of isolates	Source	Country	Year	Sequence Type (ST)	Genotypes [1]	Methodology (guidelines [2])	MIC (mg/L)	MBC (mg/L)	Reference
**Biguanides**	**Chlorhexidine**	*E. faecalis*	6	Environment (hospital sewage)	Portugal	2001-2002	6, 16, 21, 35, 49, 206		BM (CLSI)	2 - 8	4 - 16	Pereira et al., 2022
		E. faecalis	34	Environment (hospital sewage)	Iran	2018-2020			AD (CLSI)	0.5 - 8		Kheljan et al., 2022
		E. faecalis	4	Environment (urban wastewater treatment plant)	Tunisia	2014-2015	4, 23, 86, 117		BM (CLSI)	4 - 8	16	Pereira et al., 2022
		E. faecalis	21	Environment (municipal sewage)	Iran	2018-2020			AD (CLSI)	2 - 8		Kheljan et al., 2022
		E. faecalis	38	Environment (poultry sewage)	Iran	2018-2020			AD (CLSI)	0.5 - 8		Kheljan et al., 2022
		E. faecalis	29	Environment (livestock sewage)	Iran	2018-2020			AD (CLSI)	2 - 8		Kheljan et al., 2022
		E. faecalis	2	Environment (river)	Portugal	2003	1, 4		BM (CLSI)	4	16	Pereira et al., 2022
		E. faecalis	56		Spain	2001-2009			BM (CLSI)	8 - 64	32 - >64	Morrissey et al., 2014
		E. faecalis	2						AD	0.39 - 1.56		Hennessey et al., 1973
		E. faecalis	8						AD	4 - 64		Emilson et al., 1977
		E. faecalis	11						AD	2 - 32		Christensen et al., 1983
		E. faecalis	1						BM	3.9	14.3	Cowley et al., 2015
		E. faecalis SS497	1						BM	3		Kitagawa et al., 2016
		E. faecalis	1						BM	3,300		Yadav et al., 2017
		E. faecium Aus0004	1	Human infection (blood)	Australia	1998			BM (EUCAST)	4	4	Dejoies et al., 2021
		E. faecium	12	Human infection (blood)	Denmark	2004	22, 95, 178, 296, 361, 372, 697, 1277, 1279		BM (CLSI)	1 - 4	1 - 5	Alotaibi et al., 2017
		E. faecium	28	Human infection and colonization (patients without CHX bathing)	Brasil	2005-2013		*qacE* (3 isolates)	AD	1 - 32		Mendes et al., 2016
		E. faecium	20	Human infection and colonization (patients with CHX bathing)	Brasil	2005-2013			AD	4 - 32		Mendes et al., 2016
		E. faecium	37	Human infection (blood, urine, tissue)	Denmark	2012-2013	18, 80, 117, 203		BM (CLSI)	1 - 8	1 - 8	Alotaibi et al., 2017
		E. faecium	17	Human infection	Iran	2018-2020			AD (CLSI)	4 - 16		Kheljan et al., 2022
		E. faecium	45	Human infection					AD	2 - 4		Baillie et al., 1992
		E. faecium	8	Human infection					BM (CLSI)	<1 - 16		Grare et al., 2010
		E. faecium	5	Human infection (urinary, vagina, blood culture, pus, catheter, sputum)	Portugal			*qacZ*	BM (CLSI)	2 - 8		Braga et al., 2011
		E. faecium	5	Human infection (urinary, vagina, blood culture, pus, catheter, sputum)	Portugal				BM (CLSI)	4		Braga et al., 2011
		E. faecium	3	Human infection (blood)	Germany				BM (CLSI)	4 - 16		Roedel et al., 2020
		E. faecium	90	Human infection (blood cultures, swabs, urine, faeces)	Germany				BM (Vacuum Dried Biocide Microtiter Plates)	0.5 - 4	2 - 16	Roedel et al., 2020
		E. faecium	219	Human colonization (healthy humans)	Iran	2018-2020			AD (CLSI)	0.5 - 8		Kheljan et al., 2022
		E. faecium	37	Pet infection (dogs, cats)	Germany	2017-2019			BM	0.3 - 2.5		Feßler et al., 2022
		E. faecium	4	Pet infection (dogs, cats)	Portugal				BM (CLSI)	4		Braga et al., 2011
		E. faecium	22	Food chain (dust samples from breeding pig facilities)	Portugal	2008			BM (CLSI)	0.5 - 4		Braga et al., 2013

Table S1 (continued)- Distribution of cationic biocides MICs and MBCs for *Enterococcus* spp. with different epidemiological and genetic backgrounds.

Biocide class	Biocide	Species	n of isolates	Source	Country	Year	Sequence Type (ST)	Genotypes [1]	Methodology (guidelines [2])	MIC (mg/L)	MBC (mg/L)	Reference
**Biguanides**	**Chlorhexidine**	*E. faecium*	78	Food chain (pigs, broilers and cattle faeces)	Denmark				BM (CLSI)	0.5 - 8		Aarestrup and Hasman, 2004
		E. faecium CF3 1.3	1	Food chain (ceca of an adult broiler chicken)					BM (CLSI)	1		Beier et al., 2008
		E. faecium	79	Food chain (farm animal's faeces)					BM	2.5 - 250		Valenzuela et al., 2013a
		E. faecium	1	Food chain (swine meat production chain)	Italy			*qacA/B, emeA*	BM (CLSI)	14		Rizzotti et al., 2016
		E. faecium	8	Food chain (swine meat production chain)	Italy			*emeA*	BM (CLSI)	4 - 10		Rizzotti et al., 2016
		E. faecium	3	Food chain (swine meat production chain)	Italy				BM (CLSI)	8 - 10		Rizzotti et al., 2016
		E. faecium	3	Food chain (milk, cheese)	Portugal			*qacZ*	BM (CLSI)	4		Braga et al., 2011
		E. faecium	84	Food chain (traditional fermented foods)	Morocco, Spain, and Republic of Congo				BM (CLSI)	<0.1-2.5		Lavilla Lerma et al., 2014
		E. faecium	20	Food chain (fresh produce)	Spain		22, 26, 43, 46, 55, 94, 296		BM	75		Burgos et al., 2014
		E. faecium	13	Food chain (organic foods)	Spain				BM	5		Gadea et al., 2017b
		E. faecium	63	Environment (hospital sewage)	Iran	2018-2020			AD (CLSI)	0.5 - 8		Kheljan et al., 2022
		E. faecium	42	Environment (municipal sewage)	Iran	2018-2020			AD (CLSI)	1 - 16		Kheljan et al., 2022
		E. faecium	50	Environment (community waste water)	USA				BM (CLSI)	0.5 - 2		Beier et al., 2008
		E. faecium	39	Environment (poultry sewage)	Iran	2018-2020			AD (CLSI)	0.5 - 8		Kheljan et al., 2022
		E. faecium	45	Environment (livestock sewage)	Iran	2018-2020			AD (CLSI)	0.5 - 8		Kheljan et al., 2022
		E. faecium	53			1986-2009			BM (CLSI)	2 - 32	4 - 64	Morrissey et al., 2014
		E. gallinarum	1	Human infection					BM (CLSI)	2		Grare et al., 2010
		E. gallinarum	2	Food chain (dust samples from breeding pig facilities)	Portugal	2008			BM (CLSI)	1 - 2		Braga et al., 2013
		E. hirae ATCC 10541	1						BM	16		Yamamoto et al., 2016
		E. hirae	2	Human infection (urinary, vagina, blood culture, pus, catheter, sputum)	Portugal			*qacZ*	BM (CLSI)	2 - 4		Braga et al., 2011
		E. hirae	39	Food chain (dust samples from breeding pig facilities)	Portugal	2008			BM (CLSI)	0.5 - 4		Braga et al., 2013
		E. hirae	1	Food chain (milk, cheese)	Portugal			*qacZ*	BM (CLSI)	2		Braga et al., 2011
		E. raffinosus	2	Food chain (dust samples from breeding pig facilities)	Portugal	2008			BM (CLSI)	0.5		Braga et al., 2013
		E. saccharolyticus MBRG 9.16	1	Environment (domestic drain microcosm)	UK				BM	7.8	62.5	Moore et al., 2008
		E. solitarius	1	Pet infection (dogs, cats)	Portugal				BM (CLSI)	4		Braga et al., 2011

Table S1 (continued)- Distribution of cationic biocides MICs and MBCs for *Enterococcus* spp. with different epidemiological and genetic backgrounds.

Biocide class	Biocide	Species	n of isolates	Source	Country	Year	Sequence Type (ST)	Genotypes [1]	Methodology (guidelines [2])	MIC (mg/L)	MBC (mg/L)	Reference
**Biguanides**	**Chlorhexidine**	*Enterococcus* spp.	69	Human infection (blood, cerebrospinal fluid, urine, abscess, catheter tips, nasal secretions, endotracheal aspiration fluid)		2010-2011			AD (CLSI)	6 - 12		Ignak et al., 2017
		Enterococcus spp.	11	Human infection (central line–associated bloodstream infection in patients with daily CHX bathing)	USA	2012-2013			BM	2 - 16	16 - >32	Suwantarat et al., 2014
		Enterococcus spp.	19	Human infection (central line–associated bloodstream infection in patients without daily CHX bathing)	USA	2012-2013			BM	2 - 16	16 - >32	Suwantarat et al., 2014
		Enterococcus spp.	59	Human infection					BM	2.5 - 2,500		Valenzuela et al., 2013a
		Enterococcus spp.	54	Human infection					BM	16 - 128		Sobhanipoor et al., 2021
		Enterococcus spp.	18	Human colonization (skin of medical intensive care unit patients receiving CHX daily bathing)	USA	2007			BM	1 - 4		Popovich et al., 2012
		Enterococcus spp.	50	Human colonization (healthy humans' faeces)					BM	8 - 128		Sobhanipoor et al., 2021
		Enterococcus spp.	25	Food chain (dust samples from breeding pig facilities)	Portugal	2008			BM (CLSI)	<0.25 - 4		Braga et al., 2013
		Enterococcus spp.	25	Food chain (seafood)					BM	2.5 - 2,500		Valenzuela et al., 2013a
		Enterococcus spp.	40	Food chain (meat and dairy products)					BM	2.5 - 250		Valenzuela et al., 2013a
		Enterococcus spp.	22	Food chain (vegetables)					BM	2.5 - 2,500		Valenzuela et al., 2013a
		Enterococcus spp.	6	Food chain (organic foods)	Spain				BM	5		Gadea et al., 2017b
		Enterococcus spp.	47	Others (wildflowers)					BM	25 - 2,500		Valenzuela et al., 2013a
		Enterococcus spp.	107						BM (CLSI)	1.56 - 12.5		Barry et al., 1999
		Enterococcus spp.	5						AD	4 - 6		Suller and Russel, 1999
		Enterococcus spp.	32						AD	1 - 32		Mal et al., 2016
	**Polyhexamethylene biguanides**	*E. faecalis* ATCC 29212	1	Human infection (urine)	USA		30		BM (DIN:58940-7, 58940-8)	2	16	Koburger et al., 2010
		E. faecalis ATCC 29212	1	Human infection (urine)	USA		30		BM	8		Yamamoto et al., 2016
		E. faecalis ATCC 29212	1	Human infection (urine)	USA		30		BM (DIN:58940-7, 58940-8)	2		Rita et al., 2017
		E. faecalis	1	Human infection					BM (DIN:58940-7, 58940-8)	4	32	Koburger et al., 2010
		E. faecalis WIBG 1.1	1	Human infection (wound)					BM	1.8	7.3	Forbes et al., 2014
		E. faecalis	49	Pet infection (dogs, cats)	Germany	2017-2019			BM	1.25 - 80		Feßler et al., 2022
		E. faecalis	1						BM	7.8	7.8	Cowley et al., 2015
		E. faecium	37	Pet infection (dogs, cats)	Germany	2017-2019			BM	0.3 - 5		Feßler et al., 2022
		E. faecium	20	Food chain (fresh produce)	Spain		22, 26, 43, 46, 55, 94, 296		BM	8 - 60	-	Burgos et al., 2014
		E. hirae ATCC 10541	1						BM	21		Yamamoto et al., 2016
		E. saccharolyticus MBRG 9.16	1	Environment (domestic drain microcosm)	UK				BM	31.2	166	Moore et al., 2008

1 - Genes with a known or putative role in decreased susceptibility to cationic biocides.
2 - Guidelines followed in each study were included when available.
3 - Median MIC or MBC.
Abbreviations: AD, agar dilution; ATCC, American Type Culture Collection; BC, benzalkonium chloride; BM, broth microdilution; CE, cetrimide; CLSI, Clinical & Laboratory Standards Institute; CHX, chlorhexidine; CPC, cetylpyridinium chloride; DDAC, didecyldimethylammonium chloride; DIN, *Deutsches Institut fur Normung* ; EUCAST, European Committee on Antimicrobial Susceptibility Testing; MD, macrodilution; MIC, minimum inhibitory concentration; MBC, minimum bactericidal concentration; n, number; PHMB, polyhexamethylene biguanides; UK, United Kingdom; USA, United States of America.

Table S2 - Effects of *in vitro* Serial Exposure to Subinhibitory Concentrations of Cationic Biocides in *Enterococcus* spp.

Biocide class	Biocide	Species	n of isolates	Source	Exposure (n of passages when available)	Initial MIC (mg/L)	Final MIC (mg/L)	Fold MIC increase	Stability in biocide-free medium	MBC changes	Decreased biocide susceptibility	Decreased antibiotic susceptibility [1]	Other associated changes	Reference
**Quaternary Ammonium Compounds**	**Benzalkonium chloride**	*E. casseliflavus*	2	Food chain (organic foods, Spain)	Passages with increasing concentrations in Trypticase Soya Broth	0.1 - 0.2	2	10 - 20	Not stable for 20 subcultures		MIC: CPC (10 fold), CE (2 fold), CHX (100 - >100 fold), TC (100 - >100 fold), CF (>100 fold), DDAB (2 - 10 fold)	AMP		Gadea et al., 2017a
		E. durans	1	Food chain (organic foods, Spain)	Passages with increasing concentrations in Trypticase Soya Broth	0.5	2	4	Not stable for 20 subcultures		MIC: CPC (10 fold), CE (5 fold), CHX (100 fold), TC (>100 fold), CF (>100 fold), DDAB (10 fold)	AMP		Gadea et al., 2017a
		E. faecalis	2	Food chain (organic foods, Spain)	Passages with increasing concentrations in Trypticase Soya Broth	0.05 - 0.5	2.5	5 - 50	Not stable for 20 subcultures		MIC: CPC (10 - 50 fold), CHX (10 - 100 fold), TC (20 - 100 fold), CF (20 - >100 fold), DDAB (20 - 40 fold)			Gadea et al., 2017a
		E. faecalis	1		Passages with 100-fold concentration gradient (14) on Tryptone Soy Agar	2	7.8	4	Partially reversed after 14 subcultures (3.9 mg/L)	MBC increased from 3.9 mg/L to 7.8 mg/L and was stable for 14 biocide-free subcultures				Cowley et al., 2015
		E. faecium	13	Food chain (organic foods, Spain)	Passages with increasing concentrations in Trypticase Soya Broth	0.1 - 0.5	0.7 - 7	4 - 50	Not stable for 20 subcultures		MIC: CPC (5 - >100 fold), CE (2 - 20 fold), CHX (10 - 200 fold), TC (40 - >100 fold), CF (10 - >100 fold), DDAB (2 - 20 fold)	AMP, CIP, TE		Gadea et al., 2017a
		Enterococcus spp.	6	Food chain (organic foods, Spain)	Passages with increasing concentrations in Trypticase Soya Broth	0.1 - 0.5	1 - 7	4 - 35	Not stable for 20 subcultures		MIC: CPC (20 - 100 fold), CE (2 - >100 fold), CHX (40 - 100 fold), TC (50 - 100 fold), CF (10 - >100 fold), DDAB (10 fold)	AMP		Gadea et al., 2017a
	**Cetylpyridinium chloride**	*E. faecalis* SS497	1		Passages with a concentration gradient (10) in Brain Heart Infusion Broth	2	2	-	-				Increased surface hydrophobicity	Kitagawa et al., 2016
		Enterococcus spp.	4	Food chain (organic foods, Spain)	Passages with increasing concentrations in Trypticase Soya Broth	5 - 50	100 - >500	2 - >100	Stable for 20 subcultures for 1 strain		MIC: BC (>100 fold), CE (5 - 10 fold), CHX (10 - >100 fold), TC (5 - 100 fold), CF (10 - >100 fold), DDAB (2 - 10 fold)	AMP		Gadea et al., 2017a
	**Cetrimide**	*E. casseliflavus*	2	Food chain (organic foods, Spain)	Passages with increasing concentrations in Trypticase Soya Broth	5 - 10	200 - >1,000	20 - >100	Stable for 20 subcultures for 1 strain		MIC: BC (>100 fold), CPC (20 - >100 fold), CHX (40 - 100 fold), TC (10 fold), CF (>100 fold), DDAB (10 - 40 fold)	AMP		Gadea et al., 2017b
		E. faecalis WIBG 1.1	1	Human infection (wound)	Passages with 100-fold concentration gradient (10) on Mueller-Hinton Agar	12.1	14.5	1.2	Stable for 10 subcultures	MBC increased from 29 mg/L to 38.7 mg/L and continuously increased after 10 biocide-free subcultures (58 mg/L)				Forbes et al., 2014
		E. faecalis	2	Food chain (organic foods, Spain)	Passages with increasing concentrations in Trypticase Soya Broth	0.5 - 5	2 - 200	4 - 40	Not stable for 20 subcultures		MIC: BC (>100 fold), CPC (10 - >100 fold), CHX (100 fold), TC (2 fold), CF (100 - >100 fold), DDAB (10 - 20 fold)	AMP, TE		Gadea et al., 2017b

Table S2 (continued) - Effects of *in vitro* Serial Exposure to Subinhibitory Concentrations of Cationic Biocides in *Enterococcus* spp.

Biocide class	Biocide	Species	n of isolates	Source	Exposure (n of passages when available)	Initial MIC (mg/L)	Final MIC (mg/L)	Fold MIC increase	Stability in biocide-free medium	MBC changes	Decreased biocide susceptibility	Decreased antibiotic susceptibility [1]	Other associated changes	Reference
**Quaternary Ammonium Compounds**	**Cetrimide**	*E. faecium*	7	Food chain (organic foods, Spain)	Passages with increasing concentrations in Trypticase Soya Broth	0.5 - 10	>50 - 100	10 - >100	Not stable for 20 subcultures		MIC: BC (4 - >100 fold), CPC (40 - >100 fold), CHX (40 - >100 fold), TC (10 - 100 fold), CF (100 - >100 fold), DDAB (100 fold)	AMP		Gadea et al., 2017b
		Enterococcus spp.	5	Food chain (organic foods, Spain)	Passages with increasing concentrations in Trypticase Soya Broth	0.1 - 10	10 - 500	2 - >100	Stable for 20 subcultures for 1 strain		MIC: BC (100 - >100 fold), CPC (10 - >100 fold), CHX (40 - 100 fold), TC (2 - 10 fold), CF (100 - >100 fold), DDAB (10 - 40 fold)	AMP, TE		Gadea et al., 2017b
	**Didecyldimethylammonium chloride**	*E. faecalis*	4	Human	Passages with increasing concentrations (70) in Tryptone Soya Broth	0.7 - 2.7	21.9	8 - 31						Schwaiger et al., 2014
		E. faecalis	1		Passages with 100-fold concentration gradient (14) on Tryptone Soy Agar	1	2	2	Stable for 14 subcultures	MBC increased from 1 mg/L to 2 mg/L and was stable for 14 biocide-free subcultures				Cowley et al., 2015
**Biguanides**	**Chlorhexidine**	*E. casseliflavus*	3	Food chain (organic foods, Spain)	Passages with increasing concentrations in Trypticase Soya Broth	5	40 - 100	8 - 20	Stable for 20 subcultures for 1 strain		MIC: BC (30 - >100 fold), CPC (6 - 40 fold), CE (6 - 60 fold), TC (>100 fold), CF (20 - >30 fold), DDAB (2 - 6 fold)	IPM, TE		Gadea et al., 2017b
		E. durans	1	Food chain (organic foods, Spain)	Passages with increasing concentrations in Trypticase Soya Broth	5	50	10	Not stable for 20 subcultures		MIC: BC (>100 fold), CPC (20 fold), CE (>100 fold), TC (10 fold), CF (8 fold), DDAB (16 fold)	AMP, IPM		Gadea et al., 2017b
		E. faecalis ATCC 4083	1		Passages with a concentration gradient (10) in Brain Heart Infusion Broth	3.9	15.6	4	-				-	Wang et al., 2017
		E. faecalis WIBG 1.1	1	Human infection (wound)	Passages with 100-fold concentration gradient (10) on Mueller-Hinton Agar	3.6	24.2	7	Reversed after 10 subcultures	MBC increased from 26.6 mg/L to 58 mg/L and partially reversed after 10 biocide-free subcultures (29 mg/L)				Forbes et al., 2014
		E. faecalis	1	Food chain (organic foods, Spain)	Passages with increasing concentrations in Trypticase Soya Broth	5	50	10	Not stable for 20 subcultures		MIC: BC (80 fold), CPC (16 fold), CE (>100 fold), TC (2 fold), CF (30 fold), DDAB (8 fold)	IPM		Gadea et al., 2017b
		E. faecalis SS497	1		Passages with a concentration gradient (10) in Brain Heart Infusion Broth	3	11	4	-				Increased surface hydrophobicity	Kitagawa et al., 2016
		E. faecalis	1		Passages with 100-fold concentration gradient (14) on Tryptone Soy Agar	3.9	7.8	2	Continuously increased after 14 subcultures (15.6 mg/L)	MBC increased from 14.3 mg/L to 31.3 mg/L and was stable for 14 biocide-free subcultures				Cowley et al., 2015
		E. faecium	9	Food chain (organic foods, Spain)	Passages with increasing concentrations in Trypticase Soya Broth	5	10 - 80	2 - 16	Stable for 20 subcultures for 1 strain		MIC: BC (10 - >100 fold), CPC (5 - 30 fold), CE (4 - >100 fold), TC (2 - >100 fold), CF (4 - 30 fold), DDAB (4 - 8 fold)	AMP, IPM, TE		Gadea et al., 2017b

Table S2 (continued) - Effects of *in vitro* Serial Exposure to Subinhibitory Concentrations of Cationic Biocides in *Enterococcus* spp.

Biocide class	Biocide	Species	n of isolates	Source	Exposure (n of passages when available)	Initial MIC (mg/L)	Final MIC (mg/L)	Fold MIC increase	Stability in biocide-free medium	MBC changes	Decreased biocide susceptibility	Decreased antibiotic susceptibility [1]	Other associated changes	Reference
**Biguanides**	**Chlorhexidine**	*E. saccharolyticus* MBRG 9.16	1	Environment (domestic drain microcosm, UK)	Passages with 100-fold concentration gradient (14) on R2A agar	7.8	1.9	-	-	MBC decreased from 62.5 mg/L to 15.6 mg/L				Moore et al., 2008
		Enterococcus spp.	6	Food chain (organic foods, Spain)	Passages with increasing concentrations in Trypticase Soya Broth	5	10 - 50	2 - 10	Not stable for 20 subcultures		MIC: BC (30 - >100 fold), CPC (5 - 20 fold), CE (6 - 60 fold), TC (3 - 15 fold), CF (5 - 30 fold), DDAB (2 - 6 fold)	IPM, TE		Gadea et al., 2017b
	**Polyhexamethylene biguanides**	*E. faecalis* WIBG 1.1	1	Human infection (wound)	Passages with 100-fold concentration gradient (10) on Mueller-Hinton Agar	1.8	14.5	7	Partially reversed after 10 subcultures (9.7 mg/L)	MBC increased from 7.3 mg/L to 29 mg/L and reversed after 10 biocide-free subcultures				Forbes et al., 2014
		E. faecalis	1		Passages with 100-fold concentration gradient (14) on Tryptone Soy Agar	7.8	31.3	4	Partially reversed after 14 subcultures (15.6 mg/L)	MBC increased from 7.8 mg/L to 125 mg/L and partially reversed after 14 biocide-free subcultures (15.6 mg/L)				Cowley et al., 2015
		E. saccharolyticus MBRG 9.16	1	Environment (domestic drain microcosm, UK)	Passages with 100-fold concentration gradient (14) on R2A agar	31.2	20.8	-	-	MBC increased from 166 mg/L to 208 mg/L				Moore et al., 2008

1 - Among the antibiotics tested in each study that *Enterococcus* spp. are not intrinsically resistant to.

Abbreviations: AMP, ampicillin; ATCC, American Type Culture Collection; BC, benzalkonium chloride; CE, cetrimide; CF, hexachlorophene [2,20-methylenebis(3,4,6-trichlorophenol)]; CHX, chlorhexidine; CIP, ciprofloxacin; CPC, cetylpyridinium chloride; DDAB, didecyldimethylammonium bromide; DDAC, didecyldimethylammonium chloride; IPM, imipenem; MIC, minimum inhibitory concentration; MBC, minimum bactericidal concentration; n, number; PHMB, polyhexamethylene biguanides; TC, triclosan; TE, tetracycline; UK, United Kingdom.

Table S3 - Prevalence of genes with a known or putative role in decreased susceptibility to cationic biocides among collections of *Enterococcus* spp. with different epidemiological backgrounds.

Gene	Species	n of isolates	Source	Country	Year	Percentage of gene-carrying isolates (n of positive isolates)	Reference
qacA/B	*E. casseliflavus*	3	Food chain (organic foods)			0 (0)	Fernández-Fuentes et al., 2014
	E. durans	1	Food chain (organic foods)			0 (0)	Fernández-Fuentes et al., 2014
	E. faecalis	16	Human infection (pediatric bloodstream)	USA	2016-2017	43.8 (7)	Sommer et al., 2019
	E. faecalis	222	Human infection, Human colonization (healthy humans), Environment (hospital, municipal, livestock and poultry wastewater)	Iran	2018-2020	19.4 (43)	Kheljan et al., 2022
	E. faecalis	585	Human infection, Human colonization (hospitalized patients' faeces), Food chain (milk and dairy products, meat products, faeces of farm animals)	Germany		0.34 (2)	Bischoff et al., 2012
	E. faecalis	39	Human infection, Human colonization (hospitalized patients and outpatients' faeces), Food chain (milk and dairy products, meat products, faeces of farm animals)	Germany		5.12 (2, same isolates previously reported by Bischoff, 2012)	Schwaiger et al., 2014
	E. faecalis	2	Food chain (organic foods)			0 (0)	Fernández-Fuentes et al., 2014
	E. faecalis	9	Food chain (swine meat production chain)	Italy		0 (0)	Rizzotti et al., 2016
	E. faecium	37	Human infection, Human colonization, Food chain (food-animal production settings and food of animal origin), Wild birds, Aquatic environment	Portugal, Tunisia, Angola, Spain	1998-2016	0 (0)	Duarte et al., 2019
	E. faecium	48	Human infection, Human colonization	Brazil	2005-2009	0 (0)	Mendes et al., 2016
	E. faecium	3	Human infection (pediatric bloodstream)	USA	2016-2017	0 (0)	Sommer et al., 2019
	E. faecium	425	Human infection, Human colonization (healthy humans' faeces), Environment (hospital, municipal livestock and poultry wastewater)	Iran	2018-2021	10.3 (44)	Kheljan et al., 2022
	E. faecium	7	Human infection, Human colonization (hospitalized patients and outpatients' faeces)	Germany		0 (0)	Schwaiger et al., 2014
	E. faecium	13	Food chain (organic foods)			0 (0)	Fernández-Fuentes et al., 2014
	E. faecium	12	Food chain (swine meat production chain)	Italy		8.3 (1)	Rizzotti et al., 2016
	Enterococcus spp.	918	Human infection, Human colonization (healthy humans' faeces), Food chain (piggeries, aquaculture, animal/vegetable food products), Environment (hospital sewage, river)	Portugal	1996–2012	0 (0)	Silveira et al., 2015
	Enterococcus spp.	210	Human infection, Human colonization, Food chain (food-animal production settings, meat of animal origin, and other food products), Pets, Wild birds, Aquatic environment	Portugal, Tunisia, Angola, Brazil, Spain, Germany, Canada, USA	1996–2020	0 (0)	Pereira et al., 2023
	Enterococcus spp.	69	Human infection		2010-2011	0 (0)	Ignak et al., 2017
	Enterococcus spp.	54	Human infection			0 (0)	Sobhanipoor et al., 2021
	Enterococcus spp.	50	Human colonization (healthy humans' faeces)			0 (0)	Sobhanipoor et al., 2021
	Enterococcus spp.	6	Food chain (organic foods)			0 (0)	Fernández-Fuentes et al., 2014
qacC (smr, ebr, qacD)	*E. casseliflavus*	3	Food chain (organic foods)			0 (0)	Fernández-Fuentes et al., 2014
	E. durans	1	Food chain (organic foods)			0 (0)	Fernández-Fuentes et al., 2014
	E. faecalis	16	Human infection (pediatric bloodstream)	USA	2016-2017	25 (4)	Sommer et al., 2019
	E. faecalis	585	Human infection, Human colonization (hospitalized patients' faeces), Food chain (milk and dairy products, meat products, faeces of farm animals)	Germany		0.34 (2)	Bischoff et al., 2012
	E. faecalis	39	Human infection, Human colonization (hospitalized patients and outpatients' faeces), Food chain (milk and dairy products, meat products, faeces of farm animals)	Germany		5.12 (2, same isolates previously reported by Bischoff, 2012)	Schwaiger et al., 2014
	E. faecalis	2	Food chain (organic foods)			0 (0)	Fernández-Fuentes et al., 2014
	E. faecalis	9	Food chain (swine meat production chain)	Italy		0 (0)	Rizzotti et al., 2016

Table S3 (continued) - Prevalence of genes with a known or putative role in decreased susceptibility to cationic biocides among collections of *Enterococcus* spp. with different epidemiological backgrounds.

Gene	Species	n of isolates	Source	Country	Year	Percentage of gene-carrying isolates (n of positive isolates)	Reference
qacC (smr, ebr, qacD)	*E. faecium*	37	Human infection, Human colonization, Food chain (food-animal production settings and food of animal origin), Wild birds, Aquatic environment	Portugal, Tunisia, Angola, Spain	1998-2016	0 (0)	Duarte et al., 2019
	E. faecium	3	Human infection (pediatric bloodstream)	USA	2016-2017	0 (0)	Sommer et al., 2019
	E. faecium	7	Human infection, Human colonization (hospitalized patients and outpatients' faeces)	Germany		0 (0)	Schwaiger et al., 2014
	E. faecium	13	Food chain (organic foods)			0 (0)	Fernández-Fuentes et al., 2014
	E. faecium	12	Food chain (swine meat production chain)	Italy		0 (0)	Rizzotti et al., 2016
	Enterococcus spp.	918	Human infection, Human colonization (healthy humans' faeces), Food chain (piggeries, aquaculture, animal/vegetable food products), Environment (hospital sewage, river)	Portugal	1996–2012	0 (0)	Silveira et al., 2015
	Enterococcus spp.	210	Human infection, Human colonization, Food chain (food-animal production settings, meat of animal origin, and other food products), Pets, Wild birds, Aquatic environment	Portugal, Tunisia, Angola, Brazil, Spain, Germany, Canada, USA	1996–2020	0 (0)	Pereira et al., 2023
	Enterococcus spp.	69	Human infection		2010-2011	0 (0)	Ignak et al., 2017
	Enterococcus spp.	54	Human infection			0 (0)	Sobhanipoor et al., 2021
	Enterococcus spp.	50	Human colonization (healthy humans' faeces)			0 (0)	Sobhanipoor et al., 2021
	Enterococcus spp.	6	Food chain (organic foods)			0 (0)	Fernández-Fuentes et al., 2014
qacE	*E. casseliflavus*	3	Food chain (organic foods)			0 (0)	Fernández-Fuentes et al., 2014
	E. durans	1	Food chain (organic foods)			0 (0)	Fernández-Fuentes et al., 2014
	E. faecalis	45	Human infection	Japan	1996	0 (0)	Kazama et al., 1998
	E. faecalis	2	Food chain (organic foods)			0 (0)	Fernández-Fuentes et al., 2014
	E. faecalis	9	Food chain (swine meat production chain)	Italy		0 (0)	Rizzotti et al., 2016
	E. faecium	48	Human infection, Human colonization	Brazil	2005-2009	10.7 (3)	Mendes et al., 2016
	E. faecium	13	Food chain (organic foods)			0 (0)	Fernández-Fuentes et al., 2014
	E. faecium	12	Food chain (swine meat production chain)	Italy		0 (0)	Rizzotti et al., 2016
	Enterococcus spp.	6	Food chain (organic foods)			0 (0)	Fernández-Fuentes et al., 2014
qacEΔ1	*E. casseliflavus*	3	Food chain (organic foods)			0 (0)	Fernández-Fuentes et al., 2014
	E. durans	1	Food chain (organic foods)			0 (0)	Fernández-Fuentes et al., 2014
	E. faecalis	45	Human infection	Japan	1996	20 (9)	Kazama et al., 1998
	E. faecalis	222	Human infection, Human colonization (healthy humans' faeces), Environment (hospital, municipal livestock and poultry wastewater)	Iran	2018-2021	19.8 (44)	Kheljan et al., 2022
	E. faecalis	585	Human infection, Human colonization (hospitalized patients' faeces), Food chain (milk and dairy products, meat products, faeces of farm animals)	Germany		0 (0)	Bischoff et al., 2012
	E. faecalis	39	Human infection, Human colonization (hospitalized patients and outpatients' faeces), Food chain (milk and dairy products, meat products, faeces of farm animals)	Germany		0 (0)	Schwaiger et al., 2014
	E. faecalis	2	Food chain (organic foods)			0 (0)	Fernández-Fuentes et al., 2014
	E. faecalis	9	Food chain (swine meat production chain)	Italy		0 (0)	Rizzotti et al., 2016
	E. faecium	37	Human infection, Human colonization, Food chain (food-animal production settings and food of animal origin), Wild birds, Aquatic environment	Portugal, Tunisia, Angola, Spain	1998-2016	0 (0)	Duarte et al., 2019
	E. faecium	425	Human infection, Human colonization (healthy humans' faeces), Environment (hospital, municipal livestock and poultry wastewater)	Iran	2018-2021	17.2 (73)	Kheljan et al., 2022
	E. faecium	7	Human infection, Human colonization (hospitalized patients and outpatients' faeces)	Germany		0 (0)	Schwaiger et al., 2014
	E. faecium	13	Food chain (organic foods)			0 (0)	Fernández-Fuentes et al., 2014
	E. faecium	12	Food chain (swine meat production chain)	Italy		0 (0)	Rizzotti et al., 2016

Table S3 (continued) - Prevalence of genes with a known or putative role in decreased susceptibility to cationic biocides among collections of *Enterococcus* spp. with different epidemiological backgrounds.

Gene	Species	n of isolates	Source	Country	Year	Percentage of gene-carrying isolates (n of positive isolates)	Reference
qacEΔ1	*Enterococcus* spp.	918	Human infection, Human colonization (healthy humans' faeces), Food chain (piggeries, aquaculture, animal/vegetable food products), Environment (hospital sewage, river)	Portugal	1996–2012	0 (0)	Silveira et al., 2015
	Enterococcus spp.	54	Human infection			0 (0)	Sobhanipoor et al., 2021
	Enterococcus spp.	50	Human colonization (healthy humans' faeces)			0 (0)	Sobhanipoor et al., 2021
	Enterococcus spp.	6	Food chain (organic foods)			0 (0)	Fernández-Fuentes et al., 2014
qacG	*E. casseliflavus*	3	Food chain (organic foods)			0 (0)	Fernández-Fuentes et al., 2014
	E. durans	1	Food chain (organic foods)			0 (0)	Fernández-Fuentes et al., 2014
	E. faecalis	585	Human infection, Human colonization (hospitalized patients' faeces), Food chain (milk and dairy products, meat products, faeces of farm animals)	Germany		0 (0)	Bischoff et al., 2012
	E. faecalis	39	Human infection, Human colonization (hospitalized patients and outpatients' faeces), Food chain (milk and dairy products, meat products, faeces of farm animals)	Germany		0 (0)	Schwaiger et al., 2014
	E. faecalis	2	Food chain (organic foods)			0 (0)	Fernández-Fuentes et al., 2014
	E. faecium	37	Human infection, Human colonization, Food chain (food-animal production settings and food of animal origin), Wild birds, Aquatic environment	Portugal, Tunisia, Angola, Spain	1998-2016	0 (0)	Duarte et al., 2019
	E. faecium	7	Human infection, Human colonization (hospitalized patients and outpatients' faeces)	Germany		0 (0)	Schwaiger et al., 2014
	E. faecium	13	Food chain (organic foods)			0 (0)	Fernández-Fuentes et al., 2014
	Enterococcus spp.	918	Human infection, Human colonization (healthy humans' faeces), Food chain (piggeries, aquaculture, animal/vegetable food products), Environment (hospital sewage, river)	Portugal	1996–2012	0 (0)	Silveira et al., 2015
	Enterococcus spp.	210	Human infection, Human colonization, Food chain (food-animal production settings, meat of animal origin, and other food products), Pets, Wild birds, Aquatic environment	Portugal, Tunisia, Angola, Brazil, Spain, Germany, Canada, USA	1996–2020	0 (0)	Pereira et al., 2023
	Enterococcus spp.	69	Human infection		2010-2011	0 (0)	Ignak et al., 2017
	Enterococcus spp.	54	Human infection			0 (0)	Sobhanipoor et al., 2021
	Enterococcus spp.	50	Human colonization (healthy humans' faeces)			0 (0)	Sobhanipoor et al., 2021
	Enterococcus spp.	6	Food chain (organic foods)			0 (0)	Fernández-Fuentes et al., 2014
qacH	*E. casseliflavus*	3	Food chain (organic foods)			0 (0)	Fernández-Fuentes et al., 2014
	E. durans	1	Food chain (organic foods)			0 (0)	Fernández-Fuentes et al., 2014
	E. faecalis	585	Human infection, Human colonization (hospitalized patients' faeces), Food chain (milk and dairy products, meat products, faeces of farm animals)	Germany		0 (0)	Bischoff et al., 2012
	E. faecalis	39	Human infection, Human colonization (hospitalized patients and outpatients' faeces), Food chain (milk and dairy products, meat products, faeces of farm animals)	Germany		0 (0)	Schwaiger et al., 2014
	E. faecalis	2	Food chain (organic foods)			0 (0)	Fernández-Fuentes et al., 2014
	E. faecium	7	Human infection, Human colonization (hospitalized patients and outpatients' faeces)	Germany		0 (0)	Schwaiger et al., 2014
	E. faecium	13	Food chain (organic foods)			0 (0)	Fernández-Fuentes et al., 2014
	Enterococcus spp.	918	Human infection, Human colonization (healthy humans' faeces), Food chain (piggeries, aquaculture, animal/vegetable food products), Environment (hospital sewage, river)	Portugal	1996–2012	0 (0)	Silveira et al., 2015
	Enterococcus spp.	69	Human infection		2010-2011	0 (0)	Ignak et al., 2017
	Enterococcus spp.	6	Food chain (organic foods)			0 (0)	Fernández-Fuentes et al., 2014

Table S3 (continued) - Prevalence of genes with a known or putative role in decreased susceptibility to cationic biocides among collections of *Enterococcus* spp. with different epidemiological backgrounds.

Gene	Species	n of isolates	Source	Country	Year	Percentage of gene-carrying isolates (n of positive isolates)	Reference
qacJ	*E. casseliflavus*	3	Food chain (organic foods)			0 (0)	Fernández-Fuentes et al., 2014
	E. durans	1	Food chain (organic foods)			0 (0)	Fernández-Fuentes et al., 2014
	E. faecalis	585	Human infection, Human colonization (hospitalized patients' faeces), Food chain (milk and dairy products, meat products, faeces of farm animals)	Germany		0 (0)	Bischoff et al., 2012
	E. faecalis	39	Human infection, Human colonization (hospitalized patients and outpatients' faeces), Food chain (milk and dairy products, meat products, faeces of farm animals)	Germany		0 (0)	Schwaiger et al., 2014
	E. faecalis	8	Food chain (raw processed beef meat)	South Africa	2015-2016	12.5 (1)	Matle et al., 2023
	E. faecalis	2	Food chain (organic foods)			0 (0)	Fernández-Fuentes et al., 2014
	E. faecium	37	Human infection, Human colonization, Food chain (food-animal production settings and food of animal origin), Wild birds, Aquatic environment	Portugal, Tunisia, Angola, Spain	1998-2016	0 (0)	Duarte et al., 2019
	E. faecium	7	Human infection, Human colonization (hospitalized patients and outpatients' faeces)	Germany		0 (0)	Schwaiger et al., 2014
	E. faecium	13	Food chain (organic foods)			0 (0)	Fernández-Fuentes et al., 2014
	Enterococcus spp.	210	Human infection, Human colonization, Food chain (food-animal production settings, meat of animal origin, and other food products), Pets, Wild birds, Aquatic environment	Portugal, Tunisia, Angola, Brazil, Spain, Germany, Canada, USA	1996–2020	0 (0)	Pereira et al., 2023
	Enterococcus spp.	69	Human infection		2010-2011	0 (0)	Ignak et al., 2017
	Enterococcus spp.	54	Human infection			0 (0)	Sobhanipoor et al., 2021
	Enterococcus spp.	50	Human colonization (healthy humans' faeces)			0 (0)	Sobhanipoor et al., 2021
	Enterococcus spp.	6	Food chain (organic foods)			0 (0)	Fernández-Fuentes et al., 2014
qacZ	*E. faecalis*	39	Human infection, Human colonization (hospitalized patients and outpatients' faeces), Food chain (milk and dairy products, meat products, faeces of farm animals)	Germany		0 (0)	Schwaiger et al., 2014
	E. faecalis	8	Food chain (raw processed beef meat)	South Africa	2015-2016	12.5 (1)	Matle et al., 2023
	E. faecium	73	Human infection, Human colonization (hospitalized patients and outpatients' faeces)	Germany		0 (0)	Schwaiger et al., 2014
	E. faecium	37	Human infection, Human colonization, Food chain (food-animal production settings and food of animal origin), Wild birds, Aquatic environment	Portugal, Tunisia, Angola, Spain	1998-2016	2.7 (1, same isolate previously reported by Silveira 2015)	Duarte et al., 2019
	Enterococcus spp.	918	Human infection, Human colonization (healthy humans' faeces), Food chain (piggeries, aquaculture, animal/vegetable food products), Environment (hospital sewage, river)	Portugal	1996–2012	0.11 (1)	Silveira et al., 2015
	Enterococcus spp.	210	Human infection, Human colonization, Food chain (food-animal production settings, meat of animal origin, and other food products), Pets, Wild birds, Aquatic environment	Portugal, Tunisia, Angola, Brazil, Spain, Germany, Canada, USA	1996–2020	0.48 (1, same isolate previously reported by Silveira 2015)	Pereira et al., 2023
	Enterococcus spp.	54	Human infection			0 (0)	Sobhanipoor et al., 2021
	Enterococcus spp.	50	Human colonization (healthy humans' faeces)			0 (0)	Sobhanipoor et al., 2021
	Enterococcus spp.	73	Human infection (urinary, vagina, blood culture, pus, catheter and sputum), Pet infection (dogs and cats), Food chain (milk and cheese)			52 (38)	Braga et al., 2011
qrg	*E. faecium*	37	Human infection, Human colonization, Food chain (food-animal production settings and food of animal origin), Wild birds, Aquatic environment	Portugal, Tunisia, Angola, Spain	1998-2016	0 (0)	Duarte et al., 2019

Table S3 (continued) - Prevalence of genes with a known or putative role in decreased susceptibility to cationic biocides among collections of *Enterococcus* spp. with different epidemiological backgrounds.

Gene	Species	n of isolates	Source	Country	Year	Percentage of gene-carrying isolates (n of positive isolates)	Reference
qrg	*Enterococcus* spp.	210	Human infection, Human colonization, Food chain (food-animal production settings, meat of animal origin, and other food products), Pets, Wild birds, Aquatic environment	Portugal, Tunisia, Angola, Brazil, Spain, Germany, Canada, USA	1996–2020	0 (0)	Pereira et al., 2023
	Enterococcus spp.	918	Human infection, Human colonization (healthy humans' faeces), Food chain (piggeries, aquaculture, animal/vegetable food products), Environment (hospital sewage, river)	Portugal	1996–2012	0 (0)	Silveira et al., 2015
bcrABC	*Enterococcus* spp.	210	Human infection, Human colonization, Food chain (food-animal production settings, meat of animal origin, and other food products), Pets, Wild birds, Aquatic environment	Portugal, Tunisia, Angola, Brazil, Spain, Germany, Canada, USA	1996–2020	0 (0)	Pereira et al., 2023
oqxAB [1]	*E. faecalis*	51	Food chain (swine manure)	China	2010-2012	86.3 (44) *oqxA*+<br>70.6 (36) *oqxB*+	Yuan et al., 2018
	E. faecium	36	Food chain (swine manure)	China	2010-2012	69.4 (25) *oqxA*+<br>58.3 (21) *oqxB*+	Yuan et al., 2018
	E. faecium	120	Human infection (urine)	China	2017-2018	1.7 (2) *oqxA*+<br>0 (0) *oqxB*+	Zhang et al., 2021
	Enterococcus spp.	210	Human infection, Human colonization, Food chain (food-animal production settings, meat of animal origin, and other food products), Pets, Wild birds, Aquatic environment	Portugal, Tunisia, Angola, Brazil, Spain, Germany, Canada, USA	1996–2020	0 (0)	Pereira et al., 2023
	Enterococcus spp.	30	Human infection			0 (0)	Wong et al., 2015
	Enterococcus spp.	54	Human infection			0 (0)	Sobhanipoor et al., 2021
	Enterococcus spp.	7	Human colonization (healthy humans' faeces)			0 (0)	Hao et al., 2013
	Enterococcus spp.	50	Human colonization (healthy humans' faeces)			0 (0)	Sobhanipoor et al., 2021
emeA	*E. casseliflavus*	3	Food chain (organic foods)			0 (0)	Fernández-Fuentes et al., 2014
	E. durans	1	Food chain (organic foods)			0 (0)	Fernández-Fuentes et al., 2014
	E. faecalis	38	Human infection	China	2010-2012	26.3 (10)	Jia et al., 2014
	E. faecalis	26	Human infection	China	2011-2015	100 (26)	Chen et al., 2018
	E. faecalis	222	Human infection, Human colonization (healthy humans' faeces), Environment (hospital, municipal livestock and poultry wastewater)	Iran	2018-2021	42.8 (95)	Kheljan et al., 2022
	E. faecalis	78	Human infection (blood, urine), Human colonization (faeces, oral cavity), Food chain (dairy products), Water, Others			100 (78)	He et al., 2018
	E. faecalis	22	Human infection (urinary tract)	Iran		100 (22)	Esfahani et al., 2020
	E. faecalis	42	Human, Animal, Food chain, Environment			100 (42)	Panthee et al., 2021
	E. faecalis	369	Food chain (healthy chickens and pigs faeces)	Taiwan	2006-2007	100 (369)	Kuo et al., 2009
	E. faecalis	47	Food chain (commercial beef processing facility and retail ground beef)	Canada	2014-2016	100 (47)	Holman et al., 2021
	E. faecalis	28	Food chain (retail chicken meat)	South Korea	2016-2017	100 (28)	Kim et al., 2019
	E. faecalis	93	Food chain (retail meat)	South Korea	2016-2018	100 (93)	Choi and Choi, 2017
	E. faecalis	2	Food chain (organic foods)			0 (0)	Fernández-Fuentes et al., 2014
	E. faecalis	9	Food chain (swine meat production chain)	Italy		55.6 (5)	Rizzotti et al., 2016
	E. faecalis	42	Human, Animal, Food chain, Environment			100 (42)	Panthee et al., 2021
	E. faecalis	8	Food chain (raw processed beef meat)	South Africa	2015-2016	100 (8)	Matle et al., 2023
	E. faecium	62	Human infection	China	2010-2013	72.6 (45)	Jia et al., 2014
	E. faecium	120	Human infection (urine)	China	2017-2018	10 (12)	Zhang et al., 2021
	E. faecium	88	Human infection	Iran	2017-2018	54,5 (48)	Mirzaii et al., 2023
	E. faecium	425	Human infection, Human colonization (healthy humans' faeces), Environment (hospital, municipal livestock and poultry wastewater)	Iran	2018-2021	27.8 (118)	Kheljan et al., 2022
	E. faecium	1	Food chain (retail chicken meat)	South Korea	2016-2018	0 (0)	Kim et al., 2019
	E. faecium	13	Food chain (organic foods)			0 (0)	Fernández-Fuentes et al., 2014
	E. faecium	12	Food chain (swine meat production chain)	Italy		66.7 (8)	Rizzotti et al., 2016

Table S3 (continued) - Prevalence of genes with a known or putative role in decreased susceptibility to cationic biocides among collections of *Enterococcus* spp. with different epidemiological backgrounds.

Gene	Species	n of isolates	Source	Country	Year	Percentage of gene-carrying isolates (n of positive isolates)	Reference
emeA	*Enterococcus* spp.	54	Human infection			57.4 (31)	Sobhanipoor et al., 2021
	Enterococcus spp.	50	Human colonization (healthy humans' faeces)			32 (16)	Sobhanipoor et al., 2021
	Enterococcus spp.	19	Chicken litter and soil samples	South Africa	2018-2019	21 (4)	Fatoba et al., 2022
	Enterococcus spp.	6	Food chain (organic foods)			0 (0)	Fernández-Fuentes et al., 2014
efrAB	*E. casseliflavus*	3	Food chain (organic foods)			0 (0) *efrA* +<br>33.3 (1) *efrB* +	Fernández-Fuentes et al., 2014
	E. durans	1	Food chain (organic foods)			0 (0) *efrA* +<br>100 (1) *efrB* +	Fernández-Fuentes et al., 2014
	E. faecalis	80	Human infection (outpatients' urinary tract infections)	Iran	2014-2015	100 (80)	Shiadeh et al., 2019
	E. faecalis	22	Human infection (urinary tract)	Iran		100 (22)	Esfahani et al., 2020
	E. faecalis	78	Human infection (blood, urine), Human colonization (faeces, oral cavity), Food chain (dairy products), Water, Others			100 (78)	He et al., 2018
	E. faecalis	42	Human, Animal, Food chain, Environment			100 (42)	Panthee et al., 2021
	E. faecalis	47	Food chain (commercial beef processing facility and retail ground beef)	Canada	2014-2016	100 (47)	Holman et al., 2021
	E. faecalis	8	Food chain (raw processed beef meat)	South Africa	2015-2016	100 (8)	Matle et al., 2023
	E. faecalis	26	Food chain (traditional fermented foodstuffs of both animal and vegetable origins), Water	Morocco, Spain, Republic of Congo		96 (25)	Valenzuela et al., 2013b
	E. faecalis	8	Food chain (traditional fermented foods)	Morocco, Spain, Republic of Congo		100 (8)	Lavilla Lerma et al., 2014
	E. faecalis	2	Food chain (organic foods)			50 (1) *efrAB* +<br>50 (1) *efrB* +	Fernández-Fuentes et al., 2014
	E. faecium	120	Human infection (urine)	China	2017-2018	10 (12) *efrA* +<br>15.8 (19) *efrB* +	Zhang et al., 2021
	E. faecium	88	Human infection (blood, urine, wound, tracheal, fluids, sonde urinaire, sputum, fistula)	Iran	2017-2018	51 (45)	Mirzaii et al., 2023
	E. faecium	30	Food chain (traditional fermented foodstuffs of both animal and vegetable origins), Water	Morocco, Spain, Republic of Congo		13 (4)	Valenzuela et al., 2013b
	E. faecium	49	Food chain (traditional fermented foods)	Morocco, Spain, Republic of Congo		12 (6)	Lavilla Lerma et al., 2014
	E. faecium	13	Food chain (organic foods)			30.8 (4) *efrAB* +<br>7.7 (1) *efrB* +	Fernández-Fuentes et al., 2014
	Enterococcus spp.	54	Human infection			66.7 (36)	Sobhanipoor et al., 2021
	Enterococcus spp.	50	Human colonization (healthy humans' faeces)			40 (20)	Sobhanipoor et al., 2021
	Enterococcus spp.	6	Food chain (organic foods)			16.7 (1) *efrAB* +<br>33.3 (2) *efrB* +	Fernández-Fuentes et al., 2014
chlR-efrEF	*E. faecalis*	666	Human infection, Human colonization, Food chain (food-animal production settings, meat of animal origin, and other food products), Pet faeces, Aquatic environment	Portugal, Tunisia, Angola, and Brazil	1996-2020	99.8 (665)	Pereira et al., 2022
	E. faecium	37	Human infection, Human colonization, Food chain (food-animal production settings and food of animal origin), Wild birds, Aquatic environment	Portugal, Tunisia, Angola, Spain	1998-2016	100 (37)	Duarte et al., 2019
chtRS	*E. faecium*	37	Human infection, Human colonization, Food chain (food-animal production settings and food of animal origin), Wild birds, Aquatic environment	Portugal, Tunisia, Angola, Spain	1998-2016	100 (37)<br>ChtR-P102H mutation in 70 (26)	Duarte et al., 2019
	E. faecium	8	Human infection (bloodstream), Human colonization (faeces of non-hospitalized individuals)			100 (8)<br>ChtR-P102H mutation in 37.5 (3)	Guzman Prieto et al., 2017

1 - Whilst the nucleotide sequence of the *oqxA* gene is highly conserved, the sequence of *oqxB* is variable and its detection by PCR may be compromised by point mutations in primer-binding sites [183].
Abbreviations: n, number; USA, United States of America.

References

Aarestrup, F.M., and Hasman, H. (2004). Susceptibility of different bacterial species isolated from food animals to copper sulphate, zinc chloride and antimicrobial substances used for disinfection. Vet. Microbiol. 100(1-2), 83-89. doi:10.1016/j.vetmic.2004.01.013.

Alotaibi, S.M.I., Ayibiekea, A., Pedersen, A.F., Jakobsen, L., Pinholt, M., Gumpert, H., et al. (2017). Susceptibility of vancomycin-resistant and -sensitive *Enterococcus faecium* obtained from Danish hospitals to benzalkonium chloride, chlorhexidine and hydrogen peroxide biocides. J. Med. Microbiol. 66(12), 1744-1751. doi:10.1099/jmm.0.000642.

Anand, G., Ravinanthan, M., Basaviah, R., and Shetty, A.V. (2015). *In vitro* antimicrobial and cytotoxic effects of *Anacardium occidentale* and *Mangifera indica* in oral care. J. Pharm. Bioallied. Sci. 7(1), 69-74. doi:10.4103/0975-7406.148780.

Arslan, S., Ozbilge, H., Kaya, E.G., and Er, O. (2011). *In vitro* antimicrobial activity of propolis, BioPure MTAD, sodium hypochlorite, and chlorhexidine on *Enterococcus faecalis* and *Candida albicans*. Saudi Med. J. 32(5), 479-483.

Baillie, L.W., Wade, J.J., and Casewell, M.W. (1992). Chlorhexidine sensitivity of *Enterococcus faecium* resistant to vancomycin, high levels of gentamicin, or both. J. Hosp. Infect. 20(2), 127-128. doi:10.1016/0195-6701(92)90118-6.

Barry, A.L., Fuchs, P.C., and Brown, S.D. (1999). Lack of Effect of Antibiotic Resistance on Susceptibility of Microorganisms to Chlorhexidine Gluconate or Povidone Iodine. Eur. J. Clin. Microbiol. Infect. Dis. 18(12), 920-921. doi:10.1007/s100960050434.

Beier, R.C., Duke, S.E., Ziprin, R.L., Harvey, R.B., Hume, M.E., Poole, T.L., et al. (2008). Antibiotic and disinfectant susceptibility profiles of vancomycin-resistant *Enterococcus faecium* (VRE) isolated from community wastewater in Texas. Bull. Environ. Contam. Toxicol. 80(3), 188-194. doi:10.1007/s00128-007-9342-0.

Bischoff, M., Bauer, J., Preikschat, P., Schwaiger, K., Molle, G., and Holzel, C. (2012). First detection of the antiseptic resistance gene *qacA/B* in *Enterococcus faecalis*. Microb. Drug Resist. 18(1), 7-12. doi:10.1089/mdr.2011.0092.

Braga, T.M., Marujo, P.E., Pomba, C., and Lopes, M.F. (2011). Involvement, and dissemination, of the enterococcal small multidrug resistance transporter QacZ in resistance to quaternary ammonium compounds. J. Antimicrob. Chemother. 66(2), 283-286. doi:10.1093/jac/dkq460.

Braga, T.M., Pomba, C., and Lopes, M.F.S. (2013). High-level vancomycin resistant *Enterococcus faecium* related to humans and pigs found in dust from pig breeding facilities. Vet. Microbiol. 161(3), 344-349. doi:10.1016/j.vetmic.2012.07.034.

Burgos, M.J., Aguayo, M.C., Pulido, R.P., Galvez, A., and Lopez, R.L. (2014). Multilocus sequence typing and antimicrobial resistance in *Enterococcus faecium* isolates from

fresh produce. Antonie Van Leeuwenhoek 105(2), 413-421. doi:10.1007/s10482-013-0073-4.

Chen, M., Pan, H., Lou, Y., Wu, Z., Zhang, J., Huang, Y., et al. (2018). Epidemiological characteristics and genetic structure of linezolid-resistant *Enterococcus faecalis*. Infect. Drug Resist. 11, 2397-2409. doi:10.2147/idr.S181339.

Choi, M., and Choi, S. (2017). Analysis of Antimicrobial Resistance Pattern and Distribution of Multi-drug Efflux Pump Genes and Virulence Genes in *Enterococcus faecalis* Isolated from Retail Meat in Seoul. J. Food Hyg. Saf. 32(2), 135-140. doi:10.13103/jfhs.2017.32.2.135.

Christensen, K.K., Christensen, P., Dykes, A.K., Kahlmeter, G., Kurl, D.N., and Lindén, V. (1983). Chlorhexidine for prevention of neonatal colonization with group B streptococci. I. In vitro effect of chlorhexidine on group B streptococci. Eur. J. Obstet. Gynecol. Reprod. Biol. 16(3), 157-165. doi:10.1016/0028-2243(83)90095-3.

Cole, E.C., Addison, R.M., Rubino, J.R., Leese, K.E., Dulaney, P.D., Newell, M.S., et al. (2003). Investigation of antibiotic and antibacterial agent cross-resistance in target bacteria from homes of antibacterial product users and nonusers. J. Appl. Microbiol. 95(4), 664-676. doi:10.1046/j.1365-2672.2003.02022.x.

Cowley, N.L., Forbes, S., Amézquita, A., McClure, P., Humphreys, G.J., and McBain, A.J. (2015). Effects of Formulation on Microbicide Potency and Mitigation of the Development of Bacterial Insusceptibility. Appl. Environ. Microbiol. 81(20), 7330-7338. doi:10.1128/aem.01985-15.

Dejoies, L., Le Neindre, K., Reissier, S., Felden, B., and Cattoir, V. (2021). Distinct expression profiles of regulatory RNAs in the response to biocides in *Staphylococcus aureus* and *Enterococcus faecium*. Sci. Rep. 11(1), 6892. doi:10.1038/s41598-021-86376-y.

Duarte, B., Pereira, A.P., Freitas, A.R., Coque, T.M., Hammerum, A.M., Hasman, H., et al. (2019). 2CS-CHX(T) Operon Signature of Chlorhexidine Tolerance among *Enterococcus faecium* Isolates. Appl. Environ. Microbiol. 85(23), e01589-e01619. doi:10.1128/AEM.01589-19.

Emilson, C.G. (1977). Susceptibility of various microorganisms to chlorhexidine. Scand. J. Dent. Res. 85(4), 255-265. doi:10.1111/j.1600-0722.1977.tb00561.x.

Esfahani, S., Ahmadrajabi, R., Mollaei, H., and Saffari, F. (2020). Co-Incidence of Type II Topoisomerase Mutations and Efflux Expression in High Fluoroquinolone Resistant *Enterococcus faecalis* Isolated from Urinary Tract Infections. Infect. Drug Resist. 13, 553-559. doi:10.2147/idr.S237299.

Fatoba, D.O., Amoako, D.G., Akebe, A.L.K., Ismail, A., and Essack, S.Y. (2022). Genomic analysis of antibiotic-resistant *Enterococcus* spp. reveals novel enterococci strains and the spread of plasmid-borne Tet(M), Tet(L) and Erm(B) genes from chicken litter to agricultural soil in South Africa. J. Environ. Manage. 302, 114101. doi:10.1016/j.jenvman.2021.114101.

Fernández-Fuentes, M.A., Abriouel, H., Ortega Morente, E., Pérez Pulido, R., and Gálvez, A. (2014). Genetic determinants of antimicrobial resistance in Gram positive bacteria from organic foods. Int. J. Food Microbiol. 172**,** 49-56. doi:10.1016/j.ijfoodmicro.2013.11.032.

Feßler, A.T., Scholtzek, A.D., Schug, A.R., Kohn, B., Weingart, C., Hanke, D., et al. (2022). Antimicrobial and Biocide Resistance among Canine and Feline *Enterococcus faecalis*, *Enterococcus faecium, Escherichia coli, Pseudomonas aeruginosa,* and *Acinetobacter baumannii Isolates* from Diagnostic Submissions. Antibiotics (Basel) 11(2)**,** 152. doi:10.3390/antibiotics11020152.

Forbes, S., Dobson, C.B., Humphreys, G.J., and McBain, A.J. (2014). Transient and sustained bacterial adaptation following repeated sublethal exposure to microbicides and a novel human antimicrobial peptide. Antimicrob. Agents Chemother. 58(10)**,** 5809-5817. doi:10.1128/AAC.03364-14.

Gadea, R., Fernández Fuentes, M.A., Pérez Pulido, R., Gálvez, A., and Ortega, E. (2017a). Effects of exposure to quaternary-ammonium-based biocides on antimicrobial susceptibility and tolerance to physical stresses in bacteria from organic foods. Food Microbiol. 63**,** 58-71. doi:10.1016/j.fm.2016.10.037.

Gadea, R., Glibota, N., Pérez Pulido, R., Gálvez, A., and Ortega, E. (2017b). Adaptation to Biocides Cetrimide and Chlorhexidine in Bacteria from Organic Foods: Association with Tolerance to Other Antimicrobials and Physical Stresses. J. Agric. Food Chem. 65(8)**,** 1758-1770. doi:10.1021/acs.jafc.6b04650.

Grare, M., Dibama, H.M., Lafosse, S., Ribon, A., Mourer, M., Regnouf-de-Vains, J.B., et al. (2010). Cationic compounds with activity against multidrug-resistant bacteria: interest of a new compound compared with two older antiseptics, hexamidine and chlorhexidine. Clin. Microbiol. Infect. 16(5)**,** 432-438. doi:10.1111/j.1469-0691.2009.02837.x.

Guzman Prieto, A.M., Wijngaarden, J., Braat, J.C., Rogers, M.R.C., Majoor, E., Brouwer, E.C., et al. (2017). The Two-Component System ChtRS Contributes to Chlorhexidine Tolerance in *Enterococcus faecium*. Antimicrob. Agents Chemother. 61(5)**,** e02122-e02216. doi:10.1128/AAC.02122-16.

Hao, H., Guo, W., Iqbal, Z., Cheng, G., Wang, X., Dai, M., et al. (2013). Impact of cyadox on human colonic microflora in chemostat models. Regul. Toxicol. Pharmacol. 67(3)**,** 335-343. doi:10.1016/j.yrtph.2013.08.011.

He, Q., Hou, Q., Wang, Y., Li, J., Li, W., Kwok, L.-Y., et al. (2018). Comparative genomic analysis of *Enterococcus faecalis*: insights into their environmental adaptations. BMC Genomics 19(1)**,** 527. doi:10.1186/s12864-018-4887-3.

Hennessey, T.S. (1973). Some antibacterial properties of chlorhexidine. J. Periodontal Res. Suppl. 12**,** 61-67. doi:10.1111/j.1600-0765.1973.tb02166.x.

Holman, D.B., Klima, C.L., Gzyl, K.E., Zaheer, R., Service, C., Jones, T.H., and McAllister, T.A. (2021). Antimicrobial Resistance in *Enterococcus* Spp. Isolated from a Beef Processing Plant and Retail Ground Beef. Microbiol Spectr 9(3), e0198021. doi:10.1128/Spectrum.01980-21.

Ignak, S., Nakipoglu, Y., and Gurler, B. (2017). Frequency of antiseptic resistance genes in clinical staphycocci and enterococci isolates in Turkey. Antimicrob. Resist. Infect. Control 6, 88. doi:10.1186/s13756-017-0244-6.

Jia, W., Li, G., and Wang, W. (2014). Prevalence and antimicrobial resistance of *Enterococcus* species: a hospital-based study in China. Int. J. Environ. Res. Public Health 11(3), 3424-3442. doi:10.3390/ijerph110303424.

Kazama, H., Hamashima, H., Sasatsu, M., and Arai, T. (1998). Distribution of the antiseptic-resistance gene *qacEΔ1* in Gram-positive bacteria. FEMS Microbiol. Lett. 165(2), 295-299. doi:10.1016/S0378-1097(98)00291-2.

Kheljan, M., Teymorpour, R., Doghaheh, H., and Arzanlou, M. (2022). Antimicrobial Biocides Susceptibility and Tolerance-Associated Genes in *Enterococcus faecalis* and *Enterococcus faecium* Isolates Collected from Human and Environmental Sources. Curr. Microbiol. 79(6), 170. doi:10.1007/s00284-022-02858-w.

Kim, Y.B., Seo, K.W., Son, S.H., Noh, E.B., and Lee, Y.J. (2019). Genetic characterization of high-level aminoglycoside-resistant *Enterococcus faecalis* and *Enterococcus faecium* isolated from retail chicken meat. Poult. Sci. 98(11), 5981-5988. doi:10.3382/ps/pez403.

Kitagawa, H., Izutani, N., Kitagawa, R., Maezono, H., Yamaguchi, M., and Imazato, S. (2016). Evolution of resistance to cationic biocides in *Streptococcus mutans* and *Enterococcus faecalis*. J. Dent. 47, 18-22. doi:10.1016/j.jdent.2016.02.008.

Koburger, T., Hubner, N.O., Braun, M., Siebert, J., and Kramer, A. (2010). Standardized comparison of antiseptic efficacy of triclosan, PVP-iodine, octenidine dihydrochloride, polyhexanide and chlorhexidine digluconate. J. Antimicrob. Chemother. 65(8), 1712-1719. doi:10.1093/jac/dkq212.

Kõljalg, S., Naaber, P., and Mikelsaar, M. (2002). Antibiotic resistance as an indicator of bacterial chlorhexidine susceptibility. J. Hosp. Infect. 51(2), 106-113. doi:10.1053/jhin.2002.1204.

Kouidhi, B., Zmantar, T., Jrah, H., Souiden, Y., Chaieb, K., Mahdouani, K., and Bakhrouf, A. (2011). Antibacterial and resistance-modifying activities of thymoquinone against oral pathogens. Ann. Clin. Microbiol. Antimicrob. 10(1), 29. doi:10.1186/1476-0711-10-29.

Kuo, H.-C., Chou, C.-C., Chang, C.-D., Gong, S.-R., Wang, M.-H., and Chang, S.-K. (2009). Characterization of Quinolone-Resistant *Enterococcus faecalis* Isolates from Healthy Chickens and Pigs in Taiwan. J. Food Drug Anal. 17(6), 443-450. doi:10.38212/2224-6614.2576.

Lavilla Lerma, L., Benomar, N., Valenzuela, A.S., Casado Munoz Mdel, C., Galvez, A., and Abriouel, H. (2014). Role of EfrAB efflux pump in biocide tolerance and antibiotic resistance of *Enterococcus faecalis* and *Enterococcus faecium* isolated from traditional fermented foods and the effect of EDTA as EfrAB inhibitor. Food Microbiol. 44, 249-257. doi:10.1016/j.fm.2014.06.009.

Lv, S., Fan, W., and Fan, B. (2023). Enhanced in vitro antibacterial effect against *Enterococcus faecalis* by using both low-dose cetylpyridinium chloride and silver ions. BMC Oral Health 23(1), 299. doi:10.1186/s12903-023-02972-6.

Mal, P.B., Farooqi, J., Irfan, S., Hughes, M.A., and Khan, E. (2016). Reduced susceptibility to chlorhexidine disinfectant among New Delhi metallo-beta-lactamase-1 positive Enterobacteriaceae and other multidrug-resistant organisms: Report from a tertiary care hospital in Karachi, Pakistan. Indian J. Med. Microbiol. 34(3), 346-349. doi:10.4103/0255-0857.188338.

Matle, I., Atanda, A.C., Pierneef, R., Magwedere, K., and Mafuna, T. (2023). Resistome, mobilome, virulome analysis and phylogenomics of *Enterococcus faecalis* isolated from raw muscle foods of beef origin in Gauteng, South Africa. Genomics 115(6), 110742. doi:10.1016/j.ygeno.2023.110742.

Mendes, E.T., Ranzani, O.T., Marchi, A.P., Silva, M.T., Filho, J.U.A., Alves, T., et al. (2016). Chlorhexidine bathing for the prevention of colonization and infection with multidrug-resistant microorganisms in a hematopoietic stem cell transplantation unit over a 9-year period: Impact on chlorhexidine susceptibility. Medicine (Baltimore) 95(46), e5271. doi:10.1097/MD.0000000000005271.

Mirzaii, M., Alebouyeh, M., Sohrabi, M.B., Eslami, P., Fazli, M., Ebrahimi, M., et al. (2023). Antibiotic resistance assessment and multi-drug efflux pumps of *Enterococcus faecium* isolated from clinical specimens. J. Infect. Dev. Ctries 17(5), 649-655. doi:10.3855/jidc.17304.

Moore, L.E., Ledder, R.G., Gilbert, P., and McBain, A.J. (2008). *In Vitro* Study of the Effect of Cationic Biocides on Bacterial Population Dynamics and Susceptibility. Appl. Environ. Microbiol. 74(15), 4825-4834. doi:10.1128/AEM.00573-08.

Morrissey, I., Oggioni, M.R., Knight, D., Curiao, T., Coque, T., Kalkanci, A., et al. (2014). Evaluation of epidemiological cut-off values indicates that biocide resistant subpopulations are uncommon in natural isolates of clinically-relevant microorganisms. PLoS One 9(1), e86669. doi:10.1371/journal.pone.0086669.

Panthee, S., Paudel, A., Hamamoto, H., Ogasawara, A.A., Iwasa, T., Blom, J., and Sekimizu, K. (2021). Complete genome sequence and comparative genomic analysis of *Enterococcus faecalis* EF-2001, a probiotic bacterium. Genomics 113(3), 1534-1542. doi:10.1016/j.ygeno.2021.03.021.

Pereira, A.P., Antunes, P., Bierge, P., Willems, R.J.L., Corander, J., Coque, T.M., et al. (2023). Unraveling Enterococcus susceptibility to quaternary ammonium compounds: genes,

phenotypes, and the impact of environmental conditions. Microbiol. Spectr. 11(5), e0232423. doi:10.1128/spectrum.02324-23.

Pereira, A.P., Antunes, P., Willems, R., Corander, J., Coque, T.M., Peixe, L., et al. (2022). Evolution of Chlorhexidine Susceptibility and of the EfrEF Operon among *Enterococcus faecalis* from Diverse Environments, Clones, and Time Spans. Microbiol. Spectr. 10(4), e0117622. doi:10.1128/spectrum.01176-22.

Popovich, K.J., Lyles, R., Hayes, R., Hota, B., Trick, W., Weinstein, R.A., and Hayden, M.K. (2012). Relationship between chlorhexidine gluconate skin concentration and microbial density on the skin of critically ill patients bathed daily with chlorhexidine gluconate. Infect. Control Hosp. Epidemiol. 33(9), 889-896. doi:10.1086/667371.

Rita, C., Nikhil, V., and Surapaneni, S. (2017). Determination of Minimum Inhibitory Concentration (MIC) of a PolyHexamethylene Biguanide (PHMB) Solution: A Potential Root Canal Irrigant. Annu. Res. Rev. Biol. 15(2), 1-7. doi:10.9734/ARRB/2017/34141.

Rizzotti, L., Rossi, F., and Torriani, S. (2016). Biocide and antibiotic resistance of *Enterococcus faecalis* and *Enterococcus faecium* isolated from the swine meat chain. Food Microbiol. 60, 160-164. doi:10.1016/j.fm.2016.07.009.

Roedel, A., Dieckmann, R., Makarewicz, O., Hartung, A., Noll, M., Pletz, M.W., et al. (2020). Evaluation of a Newly Developed Vacuum Dried Microtiter Plate for Rapid Biocide Susceptibility Testing of Clinical *Enterococcus faecium* Isolates. Microorganisms 8(4), 551. doi:10.3390/microorganisms8040551.

Salamandane, A., Cahango, G., Muetanene, B.A., Malfeito-Ferreira, M., and Brito, L. (2023). Multidrug Resistance in Enterococci Isolated from Cheese and Capable of Producing Benzalkonium Chloride-Resistant Biofilms. Biology (Basel) 12(10), 1353. doi:10.3390/biology12101353.

Schwaiger, K., Harms, K.S., Bischoff, M., Preikschat, P., Molle, G., Bauer-Unkauf, I., et al. (2014). Insusceptibility to disinfectants in bacteria from animals, food and humans-is there a link to antimicrobial resistance? Front. Microbiol. 5, 88. doi:10.3389/fmicb.2014.00088.

Shiadeh, S.M.J., Hashemi, A., Fallah, F., Lak, P., Azimi, L., and Rashidan, M. (2019). First detection of *efrAB*, an ABC multidrug efflux pump in *Enterococcus faecalis* in Tehran, Iran. Acta Microbiol. Immunol. Hung. 66(1), 57-68. doi:10.1556/030.65.2018.016.

Sidhu, M.S., Sørum, H., and Holck, A. (2002). Resistance to quaternary ammonium compounds in food-related bacteria. Microb. Drug Resist. 8(4), 393-399. doi:10.1089/10766290260469679.

Silveira, E., Marques, P., Freitas, A.R., Mourao, J., Coque, T.M., Antunes, P., et al. (2015). A hospital sewage ST17 *Enterococcus faecium* with a transferable Inc18-like plasmid carrying genes coding for resistance to antibiotics and quaternary ammonium compounds (*qacZ*). J. Glob. Antimicrob. Resist. 3(1), 49-51. doi:10.1016/j.jgar.2014.11.005.

Sobhanipoor, M.H., Ahmadrajabi, R., Nave, H.H., and Saffari, F. (2021). Reduced Susceptibility to Biocides among Enterococci from Clinical and Non-Clinical Sources. Infect. Chemother. 53(4), 696-704. doi:10.3947/ic.2021.0090.

Sommer, L.M., Krauss, J.L., Hulten, K.G., Dunn, J.J., Kaplan, S.L., and McNeil, J.C. (2019). The prevalence of antiseptic tolerance genes among staphylococci and enterococci in a pediatric population. Infect. Control Hosp. Epidemiol. 40(3), 333-340. doi:10.1017/ice.2019.3.

Suller, M.T.E., and Russell, A.D. (1999). Antibiotic and biocide resistance in methicillin-resistant *Staphylococcus aureus* and vancomycin-resistant *Enterococcus*. J. Hosp. Infect. 43(4), 281-291. doi:10.1016/s0195-6701(99)90424-3.

Suwantarat, N., Carroll, K.C., Tekle, T., Ross, T., Maragakis, L.L., Cosgrove, S.E., and Milstone, A.M. (2014). High Prevalence of Reduced Chlorhexidine Susceptibility in Organisms Causing Central Line– Associated Bloodstream Infections. Infect. Control Hosp. Epidemiol. 35(9), 1183-1186. doi:10.1086/677628.

Touzel, R.E., Sutton, J.M., and Wand, M.E. (2016). Establishment of a multi-species biofilm model to evaluate chlorhexidine efficacy. J. Hosp. Infect. 92(2), 154-160. doi:10.1016/j.jhin.2015.09.013.

Valenzuela, A.S., Benomar, N., Abriouel, H., Cañamero, M.M., López, R.L., and Gálvez, A. (2013a). Biocide and copper tolerance in enterococci from different sources. J. Food. Prot. 76(10), 1806-1809. doi:10.4315/0362-028X.JFP-13-124.

Valenzuela, A.S., Lavilla Lerma, L., Benomar, N., Gálvez, A., Pérez Pulido, R., and Abriouel, H. (2013b). Phenotypic and molecular antibiotic resistance profile of *Enterococcus faecalis* and *Enterococcus faecium* isolated from different traditional fermented foods. Foodborne Pathog Dis 10(2), 143-149. doi:10.1089/fpd.2012.1279.

Wang, S., Wang, H., Ren, B., Li, H., Weir, M.D., Zhou, X., et al. (2017). Do quaternary ammonium monomers induce drug resistance in cariogenic, endodontic and periodontal bacterial species? Dent. Mater. 33(10), 1127-1138. doi:10.1016/j.dental.2017.07.001.

Wieland, N., Boss, J., Lettmann, S., Fritz, B., Schwaiger, K., Bauer, J., and Holzel, C.S. (2017). Susceptibility to disinfectants in antimicrobial-resistant and -susceptible isolates of *Escherichia coli, Enterococcus faecalis* and *Enterococcus faecium* from poultry-ESBL/AmpC-phenotype of *E. coli* is not associated with resistance to a quaternary ammonium compound, DDAC. J. Appl. Microbiol. 122(6), 1508-1517. doi:10.1111/jam.13440.

Wong, M.H., Chan, E.W., and Chen, S. (2015). Evolution and dissemination of OqxAB-like efflux pumps, an emerging quinolone resistance determinant among members of Enterobacteriaceae. Antimicrob. Agents Chemother. 59(6), 3290-3297. doi:10.1128/aac.00310-15.

Yadav, P., Chaudhary, S., Saxena, R.K., Talwar, S., and Yadav, S. (2017). Evaluation of Antimicrobial and Antifungal efficacy of Chitosan as endodontic irrigant against *Enterococcus faecalis* and *Candida albicans* Biofilm formed on tooth substrate. J. Clin. Exp. Dent. 9(3), e361-e367. doi:10.4317/jced.53210.

Yamamoto, M., Takami, T., Matsumura, R., Dorofeev, A., Hirata, Y., and Nagamune, H. (2016). In Vitro Evaluation of the Biocompatibility of Newly Synthesized Bis-Quaternary Ammonium Compounds with Spacer Structures Derived from Pentaerythritol or Hydroquinone. Biocontrol Sci. 21(4), 231-241. doi:10.4265/bio.21.231.

Yuan, L., Zhai, Y.J., Wu, H., Sun, H.R., He, Z.P., Wang, Y.B., et al. (2018). Identification and prevalence of RND family multidrug efflux pump *oqxAB* genes in Enterococci isolates from swine manure in China. J. Med. Microbiol. 67(6), 733-739. doi:10.1099/jmm.0.000736.

Zhang, Y., Wang, L., Zhou, C., Lin, Y., Liu, S., Zeng, W., et al. (2021). Unraveling Mechanisms and Epidemic Characteristics of Nitrofurantoin Resistance in Uropathogenic *Enterococcus faecium* Clinical Isolates. Infect. Drug Resist. 14, 1601-1611. doi:10.2147/idr.S301802.
